# The molecular features of lung cancer stem cells in dedifferentiation process-driven epigenetic alterations

**DOI:** 10.1016/j.jbc.2024.107994

**Published:** 2024-11-14

**Authors:** Valentina Masciale, Federico Banchelli, Giulia Grisendi, Anna Valeria Samarelli, Giulia Raineri, Tania Rossi, Michele Zanoni, Michela Cortesi, Sara Bandini, Paola Ulivi, Giovanni Martinelli, Franco Stella, Massimo Dominici, Beatrice Aramini

**Affiliations:** 1 Laboratory of Cellular Therapies, Department of Medical and Surgical Sciences for Children & Adults, University Hospital of Modena, Modena, Italy; 2 Department of Statistical Sciences "Paolo Fortunati", Alma Mater Studiorum- University of Bologna, Bologna, Italy; 3 Laboratory of and Respiratory Medicine, Department of Medical and Surgical Sciences for Children & Adults, University Hospital of Modena, Modena, Italy; 4 Biosciences Laboratory, IRCCS Istituto Romagnolo per lo Studio dei Tumori (IRST) "Dino Amadori", Meldola, Italy; 5 Thoracic Surgery Unit, Department of Medical and Surgical Sciences-DIMEC of the Alma Mater Studiorum, University of Bologna, G.B. Morgagni-L. Pierantoni Hospital, Forlì, Italy; 6 Division of Oncology, University Hospital of Modena and Reggio Emilia, University of Modena and Reggio Emilia, Modena, Italy

**Keywords:** cancer stem cells, lung cancer, differentiation, dedifferentiation, transdifferentiation, epigenetic, molecular pathways

## Abstract

Cancer stem cells (CSCs) may be dedifferentiated somatic cells following oncogenic processes, representing a subpopulation of cells able to promote tumor growth with their capacities for proliferation and self-renewal, inducing lineage heterogeneity, which may be a main cause of resistance to therapies. It has been shown that the “less differentiated process” may have an impact on tumor plasticity, particularly when non-CSCs may dedifferentiate and become CSC-like. Bidirectional interconversion between CSCs and non-CSCs has been reported in other solid tumors, where the inflammatory stroma promotes cell reprogramming by enhancing Wnt signaling through nuclear factor kappa B activation in association with intracellular signaling, which may induce cells' pluripotency, the oncogenic transformation can be considered another important aspect in the acquisition of “new” development programs with oncogenic features. During cell reprogramming, mutations represent an initial step toward dedifferentiation, in which tumor cells switch from a partially or terminally differentiated stage to a less differentiated stage that is mainly manifested by re-entry into the cell cycle, acquisition of a stem cell-like phenotype, and expression of stem cell markers. This phenomenon typically shows up as a change in the form, function, and pattern of gene and protein expression, and more specifically, in CSCs. This review would highlight the main epigenetic alterations, major signaling pathways and driver mutations in which CSCs, in tumors and specifically, in lung cancer, could be involved, acting as key elements in the differentiation/dedifferentiation process. This would highlight the main molecular mechanisms which need to be considered for more tailored therapies.

Lung cancer is considered one of the most debated unsolved health problems globally, representing 19.4% of all tumors, and causing death for 1.59 million people (1). In recent years, the scientific community has defined solid tumors as a mix of cells with different activities and properties (2). Although new approaches have been set for local and advanced diseases, lung cancer has a strong tendency to recur, suggesting the possible presence of a cell population that develops resistance against standard treatments (3). Cells with this peculiar molecular profile have been defined as having the “capacity to renew and regenerate themselves,” and have been termed “cancer stem cells” (CSCs) (4). The CSC hypothesis was born in the mid-19th century when Rudolph Virchow and Julius Cohnheim reported similar characteristics between different types of cancers, such as teratomas, and developed fetuses leading to the theory that cancer cells may be derived from “peculiar” cells with the capacity to self-renew and differentiate (4, 5, 6).

Bonnet and Dick were the first to identify a subpopulation of human acute myeloid leukemia (AML) cells, marked by CD34+ CD38, which were able to induce malignancy in transplanted mice (7). These cells can induce tumor development, the metastatic process, and tumor recurrence. Following this, they tried to isolate similar subpopulations from different solid organs, such as the lung. Based on the considerations related to the CSC model, normal stem cells may be considered primary tumorigenic cells, possessing the potential to activate tumorigenic survival pathways that can induce indefinite tumor cell proliferation (4, 7). The development of oncogenic mutations transforms normal stem cells into cancer cells that can expand *in vitro* as spheres and reproduce when implanted in immunodeficient mice (8, 9). Targeting stem cells expressing anti-apoptotic and drug-resistant proteins at high levels is an important treatment strategy against most lung tumors; however, such treatment has not demonstrated efficacy against cancer metastasis and relapse (10). This approach has been long investigated, highlighting the desire to identify specific markers against CSCs (11, 12, 13, 14, 15).

However, the localization of such cells in the lung has led to a debate regarding this association, as the lung epithelium has a very low proliferation rate of 0.06 to 1.3%. Some hypotheses theorize that tissue renewal may host stem cells (16). The association between cellular heterogeneity and lung structure complicates CSC identification (16, 17). Jackson *et al.* described the activation of the oncogenic KRAS by Clara cells and surfactant protein C (SPC) as a potential tumor trigger mechanism (18). Kim *et al.* reported that bronchioalveolar stem cells (BASCs, marked by a double-positive stained cell), which are quiescent in normal lungs, start to proliferate after chemical treatment or drugs (19). It is believed that BASCs can self-renew and, therefore, may develop dangerous mutations due to their potential to transform into CSCs. The ability to identify these cells in the bronchioalveolar duct junction indicates they have a high degree of tumorigenicity for stem cells (20). Adenocarcinomas (ACs) are thought to be derived from these BASCs, which develop from normal stem cells (6). Studies using single-cell sequencing to analyze patients with AML have demonstrated that normal hematopoietic stem cells present the same mutations as those found in AML blasts. Such findings are aligned with the model showing that cancer develops from sequential mutations that accumulate in adult stem cells (21, 22, 23, 24, 25, 26, 27, 28, 29, 30, 31, 32).

## CSCs molecular markers

No specific CSCs markers have yet been identified (33, 34, 35, 36) in solid tumors and, specifically in lung cancer. This is due to the significantly heterogenous nature of CSCs, which can differ due in their proliferative status (cycling or noncycling) and expression markers (37). Many proposed agents targeting CSCs have failed in clinical trials due to acting only on one subset of CSCs, such as cycling cells, and having no effect on quiescent CSCs (38, 39, 40, 41). The most common surface markers used to identify CSCs have been CD44 and CD133 (42, 43); however, recently CD166 and EpCAM have been identified (44, 45). CD44 is a transmembrane glycoprotein that binds hyaluronic acid and facilitates CSCs adhesion, differentiation, homing, and migration (46). It is considered an important molecule due to its role in CSCs identification and its potential regulatory role in cell differentiation, diffusion, and cancer development. Furthermore, CD44 expression has been noted in metaplasia, suggesting a possible role in tumor progression (46, 47).

Leung *et al.* (43) described the capacity of a CD44+ subpopulation to produce spheroids expressing stemness genes, such as octamer-binding transcription factor 4 (OCT4), sex-determining region Y-box 2 (SOX2), and NANOG, which were not found in CD44− cells (48). Moreover, CD44+ cells were found to be resistant to chemotherapy (49, 50). These characteristics of CD44+ positive cells, may explain even their resistance to chemotherapy (49, 50). Moreover, based on these findings (51), CD44 was identified as a possible stem cell marker.

The cell surface glycoprotein CD133 appears to play a role in defining a cell population that induces tumor sphere formation (52). These cells can remain undifferentiated or differentiated, resembling a population of tumor cells (52, 53). Like CD44+ cells, CD133+ cells demonstrate chemotherapy resistance (52, 54). This explains the poor long-term survival reported in several patients with non–small cell lung cancer (NSCLC). Although discussions around the role of CD133+ cells as a possible prognostic marker are new, researchers believe they may be a good predictor of cytotoxic efficacy in therapy; however, they may not represent a prognostic marker for NSCLC (54).

Another potential marker is CD166, which is activated by the leukocyte cell adhesion molecule and plays an active role in angiogenesis and differentiation (55). A subpopulation of CD166+ cells have previously demonstrated the ability to self-renew, generate primary and secondary xenograft tumors phenotypically, and mimic parental tumors (55). CD166+ cells can produce spheres *in vitro*, which may initiate tumorigenesis *in vivo*. However, a knockdown test did not show the expected results, as low CD166 expression in CSCs did not influence carcinogenesis (55, 56).

The expression of aldehyde dehydrogenase (ALDH) has been used for CSC detection in many solid tumors, such as breast, lung, and melanoma tumors (57, 58, 59). Jiang *et al.* (60) first showed ALDH1+ cells from lung cancer (60). These cells demonstrated the ability to self-renew and differentiate and exhibited multidrug resistance. The researchers also studied ALDH1 expression in 303 lung tissues and found that increased ALDH1 protein levels were strongly associated with tumor stages and grades and inversely correlated with patient survival (61, 62).

Masciale *et al.* demonstrated that the presence of CSCs resulted in spheroid formation in human lung cancer tissues extracted from patients who underwent surgery for NSCLC and were positive for the ALDH marker (63). These cells produced spheres in a serum-freecultured medium for a minimum of 3 weeks and demonstrated overexpression of *SOX-2* and *NANOG* compared to ALDH− cells (63). Cells overexpressing ALDH showed lower levels of reactive oxygen species (ROS) than differentiated cancer cells (52), which seem to be due to an NRF2-mediated increase in antioxidant enzymes, such as GPX3, SOD-2, and HO-1 (64, 65). This explains their resistance to chemotherapy and radiotherapy, both of which induce ROS production, as CSCs can upregulate antioxidant levels (64, 65). Specifically, ALDH1A1 and 3A1 enzymatic isoforms can convert drugs into nontoxic metabolites. Cells expressing ALDH1A1 appear resistant to the epidermal growth factor receptor (EGFR) tyrosine kinase inhibitor (TKI) known as gefitinib (64).

As ALDH overexpression is strongly associated with therapy resistance and CSCs self-renewal, differentiation, and oxidative stress protection, targeting enzymes may be a potential approach against cancer (66). For example, the α, β-acetylenic amino-thiol ester family are powerful multi-ALDH inhibitors used against NSCLC (64). One such ester is 4-dimethylamino-4-methyl-pent-2-thioic-S-methylester acid, which inhibits ALDH1 and ALDH3 competitively and irreversibly (67). In combination with chemotherapy, 4-dimethylamino-4-methyl-pent-2-thioic-S-methylester acid use has resulted in aldehyde and aldehyde-protein adduct accumulation without toxic side effects in animals (68). While no clinical trials have been described in the literature, promising results indicate that ALDH enzyme presence may induce toxicity in healthy tissues (69). For these reasons, scientists have shifted focus to identifying surface markers related to ALDH (70, 71). In 2020, Masciale *et al.* found a population of cells positive for both CD44 and EPCAM, similar to ALDH-high cells (45), reporting a strong correlation between ALDH-high cells and CD44+/EpCAM+ cells. Moreover, the CD44+/EpCAM+ cells demonstrated the same capacity to form spheroids *in vitro* as the ALDH-high cells and a similar level of stemness gene expression (45).

Identifying a surface marker would be more useful for defining targeted CSC treatments. Although ALDH has shown promise as a CSC marker, it is not exclusive to CSCs. Consequently, researchers have been hesitant to definitively label it as a CSC marker, and further research is needed. While progress has been made in recognizing CSC’s existence in lung cancer, identifying a specific target for intratumoral heterogeneity and high plasticity remains challenging, as both are associated with CSC phenotypic instability and cell surface marker reversion (72). Thus, for the moment, a specific approach to identify specifically lung cancer CSCs has not been found.

This review article would highlight the main epigenetic determinants in lung cancer and driver mutations in which CSCs could act as key elements in the differentiation/dedifferentiation process. These molecular features described in this review will guide the scientific community on new considerations regarding lung cancer development and progression, and they would be a font of inspiration for the setting of future perspectives on new technologies and more focused approaches against cancer.

## The oncogenic processes behind CSCs

CSCs are considered an important target for cancer treatment due to their contributions to the carcinogenetic process in several solid tumors, including differentiation, dissemination, relapse, and drug/radiation resistance (4, 73, 74) through the activation of molecular signal cascades. That control processes associated with tumorigenesis (75, 76, 77, 78). CSCs appear to be regulated by several signaling pathways, including the WNT/beta-Catenin, *NOTCH, MYC* Janus kinase/signal transducers and activators of transcription (*JAK-STAT*), PI3K/AKT/mammalian target of rapamycin (mTOR), transforming growth factor (TGF)/SMAD, and peroxisome proliferator-activated receptor pathways (75, 76). Several other key genes, such as *SOX2, NANOG, KLF4, and OCT3/4,* are involved in the maintenance of the stem cell compartment (77, 78). CSCs are also regulated by epigenetic alterations mediated *via* methylation and noncoding RNAs, which can result in the deregulation and gain of function in somatic stem cells (79). CSCs appear to share several characteristics with embryonic stem (ES) cells, such as self-renewal, proliferation, and differentiation inhibition (80), and CSCs from epithelial tumors demonstrate expression of the c-*MYC* oncogene and other factors that are crucial for their pluripotency, including *SOX2, DNMT1, CBX3, and HDAC1*. The first CSCs identified in a solid tumor were from a mammary carcinoma (81, 82). These cells possessed an EPCAM + ESA + CD44 + CD24−/low phenotype that was capable of propagating mammary tumors after low-dose injections into immunodeficient mice (83). CD44 is a cell membrane protein that plays an important role in tumor microenvironments (TMEs), specifically between cell-to-cell interactions and cell-extracellular matrix (ECM) connections (83, 84). Studies have linked CD44 to different tumor cell processes, such as adhesion, migration, and metastasis. The coexpression of CD24 characterizes a subpopulation of ALDH cells that have been found to induce cancer diffusion in immunodeficient mice (84, 85). CD44 can protect cells from apoptosis, an important feature of CSCs. CSCs support the TME which benefits their survival and growth, forming a CSC-like state (84, 85, 86).

Understanding the impact of the DNA damage has become a mainstream strategy for targeted cancer therapy; in the cellular compartment a sensing machine is activated as a response to DNA damage. Stem cells are extremely sensible to this damage. These kinds of injuries in stem cells may drive stem cells to senescence, apoptosis, or transformation, depending on the type of lesion and extent of damage. Furthermore, mutations and genetic or epigenetic changes can turn normal stem cells into CSCs (87). Though it is impossible to say with certainty that a cell with more driver mutations has a less uninvolved phenotype than normal stem cells, some authors propose that driver mutations increase the stemness of cancer cells (88, 89). Several data points (*e.g.*, KRAS in colorectal CSCs (90)) support the existence of driver mutations in CSCs.

The surface marker Sca-1, which was first used to target the (BASC), identifying tumor-propagating cells (TPCs) in genetically engineered mice models of NSCLC bearing KRAS mutation (91). It is interesting to note that this study concluded that surface markers' ability to enrich for TPCs was influenced by the genotype of the tumor; in KRAS mutant mice, tumors containing only KRAS mutations did not exhibit tumors that Sca-1 enriched for TPCs. Considering the fact that lung cancers have one of the highest mutational burdens of any cancer, this a crucial decision. According to Lau *et al.* (2014) (92), a TPC population that was also highly metastatic was subsequently refined by utilizing CD24 expression in combination with Sca-1. TPCs, which were also susceptible to Notch inhibition, were enriched for using CD24 in combination with ITGB4 and the Notch receptor expression in the same mouse model (93). These investigations employed a range of transplant methodologies, including serial transplantation, in which transplant cells are at gradually lower dilutions until there are insufficient cells to form new tumors (typically as low as 1–10 cells). These studies are the "gold standard" for demonstrating sustained self-renewal or the enrichment of a particular population in CSCs, or precisely determining the TPC frequency in the cancer cells under study. These provide more information than transplant assays or single-cell culture techniques (colony, or sphere/organoid/"tumorsphere"), which quantify the presence or function of CSCs but not the number of tumor cells that can initiate a new tumor after a single bulk injection.

The idea that lung stem cells are the cells that give rise to lung cancer is another way that stem cells and cancer interact. This question has been investigated using mouse models of lung cancer (94). As one of the most common mutations in lung AC, many different types of aggressive cancers have oncogenic KRAS mutations. Consequently, there has been a great deal of interest in the mechanisms behind KRAS oncogenesis and the cell types that are vulnerable to this kind of change. Lox-Cre recombination conditionally removes a stop codon, resulting in KRAS LSLG12D/V alleles, which in turn promotes tumor initiation in these models. Experiments that link specific gene expression to tumor initiation and lineage tracing that allows tracking of labeled cell progeny have greatly aided this strategy (94).

Over the last 10 years, there has been a lot of discussion about the alveolar type II cell (AT2) and a rare subpopulation of cells known as basioalveolar duct junction (BADJ) cells (94, 95). Operationally, "double-positive cells" are sometimes used to describe BASCs because they express both the bronchiolar Clara cell antigen 10 (CC10, CCSP) and the AT2 marker SPC (Sftpc) (95, 96). Kim *et al.* (2005) (97) demonstrated that early KRAS tumors exhibited double-positive BASC expansion, and Ventura *et al.* (2007) (98) and Dovey *et al.* (2008) (96) demonstrated that the rate of tumorigenesis in KRAS AC was impacted by the manipulation of BASC regulators, p38a and Bmi1. Tumors express SPC uniformly and primarily originate in the alveolar regions, according to research using SPC-driven Cre system to activate KRAS exclusively in SPC-expressing cells (99, 100). This suggests that AT2 cells are representative of the tumor-initiating cells (TIC). Some have proposed that a subset of AT2 cells function as stem cells and represent the true cell of origin. It is also proposed that the cell of origin is a double-positive alveolar cell.

In some cases, SPC + AT2 cells in the alveoli and CC10 + cells in the BADJ—rather than the proximal bronchiolar airways—could be progenitor AC cells bearing KRAS mutation (101, 102). Tamoxifen-induced SPC-Cre-ERT fusions drive tumors, but only alveolar lesions multiply beyond 20 cells. On the other hand, more BADJ lesions, which develop into more advanced adenomas, are produced when tumors are started with the Cre adenovirus. This depends on inflammation brought on by a viral infection, demonstrating how environmental variables affect the development of KRAS-driven tumors that start in various places.

According to Sutherland *et al.* (2014) (102), CC10 + and CC10/SPC tumors form at the BADJ, whereas SPC + tumors form in the alveoli. These tumors are caused by viruses delivering SPC and CC10 Cre. Jackson *et al.* (2005) (103) and Curtis *et al.* (2010) (91) discovered that p53 loss induced characteristics of metastasis and invasiveness, such as the upregulation of high mobility group protein A2 expression and loss of Nkx2-1 and accelerated the growth of tumors driven by SPC-Cre and CC10-Cre (104). These results align with earlier studies conducted in this field. According to KRAS models, SPC-driven tumors in the alveolar region are more advanced than P53- or BADJ-driven tumors. Additionally, these tumors were more metastatic and invasive, indicating that certain cells of origin are affected differently by oncogenic genotypes than others, a phenomenon seen in other forms of tumor (105).

Subsequent genetic mutations can alter the cell of origin once more; Axin + AT2 cells are known to be the preferred source of *KRAS, P53*, and *PRTCΙ* mutant tumors (106). Many subtypes of lung cancer are thought to develop from different pulmonary stem cells. Strengthening evidence suggests that the basal cell, the stem cell responsible for producing the upper airway epithelium, is the source of this particular histologic type of lung tumor (99, 107, 108). Lesions of squamous cell carcinoma begin in the upper airways and bronchioles. Transdifferentiated ACs from AT2 and double-positive cells have been shown to give rise to adenosquamous NSCLC, which has a histology of both squamous and ACs (109, 110). Neuroendocrine cells (111, 112), which can function as progenitor cells, account for nearly all of the origins of SCLC (113, 114). A tiny fraction is thought to have originated from tuft cells.

Lung cancer cells have been cultured by researchers in a way that favors more tumorigenic traits, like transplantation efficiency, sphere-forming ability, or *in vitro* migration properties, in an effort to find lung CSCs (115). Other tumor lineages have also been found to contain these markers. Since individual investigations rarely attempt to validate published findings from other studies, it is frequently unclear whether the cell populations being described are synonymous with, overlap with, or distinct from earlier discoveries (115, 116, 117, 118). Lung AC in KRAS; P53 mouse models has been shown to exhibit certain characteristics of CSCs (92, 93, 94). CD24 + cells, CD24 + Sca-1 + cells, and CD24 + ITGB4 + Notch^hi^ cells make up these populations (92, 93). There is proof that the phenotypes of these populations are different. For instance, tumors can spread effectively by both CD24+ and CD24 +Sca1 + cells, but CD24+ Sca1 + cells metastasize considerably more frequently. It was discovered that CD24+ ITGB4 +Notch^hi^ cells, which exhibit chemotherapy drug resistance, were enriched in culture following cisplatin treatment (93). These cells are also capable of spreading tumors.

Hence, these cells may be simulating chemo-resistant CSCs that proliferate tumors following treatment. Novel technologies (scRNA-seq) that allow for single-cell transcription analysis of rare populations will be used to address many of these questions (119, 120, 121). These will make it possible for researchers to look into the possible heterogeneity of CSCs and see if the various populations described in the literature overlap (119, 120, 121).

### Major CSC transcription factors

The ability of stem cells to self-renew and differentiate into tissue-specific cells is peculiar. Temporary overexpression of the *OCT4, SOX2, NANOG, KLF4, and MYC* embryonic transcription factors can be used to reprogram somatic cells into stem cells (122). The similarities between CSCs and stem cells are due to the expression of these embryonic transcription factors, which confer pluripotency (123). Therefore, the role of transcription factors is considered crucial for CSC development. Of these, one of the most important is OCT4, a homeodomain transcription factor of the Pit-Oct-Unc family that controls stem cell capacity for pluripotency and self-renewal and is involved in their maintenance (76, 124). An investigation into germ cell tumors (GCTs) of the testis and ovary showed that OCT4 directly controls stemness and metastasis by organizing the ECM and focal adhesions (125).

#### SOX2

SOX2 is regarded as one of the most significant transcription factors for CSCs (126, 127). It belongs to a high-mobility family of transcription factors and is crucial for the generation and maintenance of stem cells (128). Other SOX2 studies have reported that it promotes tumorigenesis and sustains the self-renewal of CSCs (129). Rodriguez-Pinilla *et al.* (130) found that elevated SOX2 expression in breast cancer cells might encourage the development of phenotypes (82) associated with poorly/undifferentiated stem cells. Moreover, SOX2 can lead to glioma formation, and SOX2-KO mice demonstrated inhibition of glioblastoma development and tumorigenicity, suggesting that this transcription factor is crucial for the preservation of self-renewal balance (130, 131). The same characteristics have been shown in osteosarcomas TICs, whereby SOX2 downregulation leads to tumorigenesis reduction *in vitro*. The loss of downregulation of SOX2 expression prevents the formation of osteosarcoma spheres (132, 133).

#### NANOG

NANOG is a differentiated homeobox (HOX) domain protein with similar characteristics to SOX2, playing a pivotal role in self-renewal and transcriptional regulation (134, 135, 136). Overexpression of NANOG has been seen in several solid human cancers, such as breast, brain, colon, and lung cancer. NANOG levels are higher in malignancies than normal tissue (137, 138). For example, high NANOG levels have been linked to lymph node positivity and Duke’s grade in individuals with colorectal cancer (76). This is in line with NANOG’s influence on sphere creation and tumor aggressiveness *in vivo*. Similarly, in gastric cancer, high levels of NANOG reduce overall survival and increase tumor dissemination. In hepatocellular carcinoma (HCC) cell lines, NANOG overexpression is correlated with advanced disease stages (III and IV TNM stages) (139). Similar results were reported in 43 patients with pancreatic cancer who had high levels of NANOG and OCT4 and demonstrated worse prognoses and very low survival. These findings indicate that NANOG is crucial for CSC regulation, self-renewal, and proliferation (140).

#### KLF4

Numerous tissues express KLF4, which is crucial to many physiological processes. KLF4 can activate or inhibit transcription through different mechanisms, depending on the target gene (141). Depending on the type of cancer, KLF4 may have an oncogenic or anticancer effect. For instance, it functions as an anticancer agent in the stomach and intestinal epithelium (142). KLF4 downregulation is seen in several solid cancers, such as liver, lung, and bladder cancer. Although KLF4 has clear anticancer activity, its role as an oncogene remains largely undiscussed (143). However, it has been demonstrated that in rat kidney cells it induces tumorigenesis. Similarly, the overexpression of KLF4 in breast cancer CSCs is correlated with an aggressive phenotype. KLF4 seems to have different functions in CSCs (144).

#### MYC

The proto-oncogenes C-MYC, N-MYC, and L-MYC are members of the MYC family (145, 146). This family codes for transcription factors that regulate the transcription of at least 15% of the entire genome (147). MYC is essential for many oncogenic processes, such as differentiation, apoptosis, proliferation, and metabolism (148, 149). MYC expression is deregulated in tumors. MYC regulates the self-renewal ability of CSCs, as MYC is a target of the Wnt/β-catenin pathway that is linked to CSC self-renewal in several solid tumors, such as colon and breast cancers and gliomas (76, 150, 151). In cancer, c-MYC aids multidrug resistance by dysregulating the transcription of specific ABC transporter genes (152). However, the C-MYC regulatory mechanism underpinning the maintenance of self-renewal and drug-resistance properties in colon CSCs is not yet understood (150). It has been demonstrated that the downregulation of c-MYC inhibits the suppression of CSC self-renewal in colon cancer xenografts (153). Furthermore, c-MYC reduction may induce chemosensitivity in colon CSCs through ABCG2 and ABCB5 downregulation (150). The MYC-mediated preservation of CSC compartments also involves the tumor suppressor p53. MYC and P53 activate cell proliferation and carcinogenesis in HCC (154). The deregulation of MYC and P53 combined with *BCL-2* and *BMI-1* overexpression and the loss of p19ARF may facilitate CSC survival and proliferation (154, 155). MYC family members act at different levels in various tumors, with c-MYC predominant in leukemia, l-MYC in hematopoietic malignancies, and n-MYC in SCLC (156). MYC inactivation causes HCC stem cells to differentiate, which leads to a decrease in tumor markers and an increase in hepatocytes, cytokeratin 8, and liver stem cell cytokeratin indicators (157, 158). Studies have shown that MYC is essential for the proliferation and invasion of glioblastoma multiforme stem cells, which in turn prevents apoptosis (159). Prostate cancer–related CSCs have been shown to have higher copy numbers of the MYC gene, suggesting a function in tumorigenesis activation (160).

### Major signaling pathways associated with CSCs

Signaling pathways play important roles in CSC maintenance and survival, regulating their self-renewal and differentiation properties and activating or repressing the tumorigenic process (75, 76). Genetic and epigenetic factors regulate these pathways (161, 162).

In particular, DNA methylation was one of the first epigenetic modifications to be identified—even before the double helix structure of DNA was discovered. DNA methyltransferase (DNMT) adds a methyl group to the cytosine fifth carbon in the DNA sequence, a process known as DNA methylation (163, 164). This results in 5-methylcytosine in the DNA sequence. The majority of these changes take place on CpG islands. DNMT1, DNMT3A, and DNMT3B are the three methyltransferases associated with methylation in humans. DNMT1 methylates hemimethylated DNA during the cell division cycle following DNA replication, while DNMT3A and DNMT3B initiate new DNA methylation (165).

According to Liu *et al.* (2015) (166), the interleukin-6 (IL-6)/JAK2/STAT pathway may downregulate P53 and P21 expression, which could increase tumor initiation, upregulate DNMT1, and encourage the growth of lung CSCs. This indicates that low expression of these genes may aid in the development of lung CSCs and that DNA hypermethylation is linked to the silencing of oncogene and differentiation genes in lung cancer. Nusse and Clevers (2017) (167) have shown that activation of the WNT signaling pathway in mouse models is associated with a higher risk of tumor initiation (167).

The development of lung adenocarcinoma may result from the aberrant activations of the Wnt/β-Catenin pathway caused by APC, LKB1, WNT inhibitor 1 (WNT-1), Disabled-2, and members of the Dickkopf (Dkk) family. The aberrant Wnt/β-Catenin pathway activations are related to these hypermethylated silencing factors. According to Zhang *et al.* (2018) (168), overexpressed G9a may influence the growth of lung cancer cells by suppressing the expression of WNT-1 through DNA hypermethylation, which would result in an aberrant WNT pathway. One secretory protein that inhibits WNT signaling is called DKK1. Park *et al.* (2012) and Han *et al.* (2017) (169, 170) demonstrated that the WNT signaling pathway facilitates the growth of lung cancer by means of the DNA hypermethylation of the DKK1 promoter.

#### Wnt signaling pathway

The Wnt pathway is multifaceted and controls important cell processes such as organogenesis, migration, fate, and polarity (171). With over 15 receptors and 19 Wnt ligands, the pathway is complex. There are two forms of the pathway: the “Wnt canonical” and “Wnt noncanonical” pathways (171, 172, 173). Specifically, in the canonical pathway, known as the Wnt/β-catenin pathway, complex receptors such as FZD-LRP5/6 are activated, consequently deregulating β-catenin (174). In the noncanonical pathway, the activation process involves FZD receptors and/or ROR1/ROR2/RYK coreceptors, leading to a consequent cascade of PCP, RTK, or Ca2+ signaling (175).

**Figure 1:**
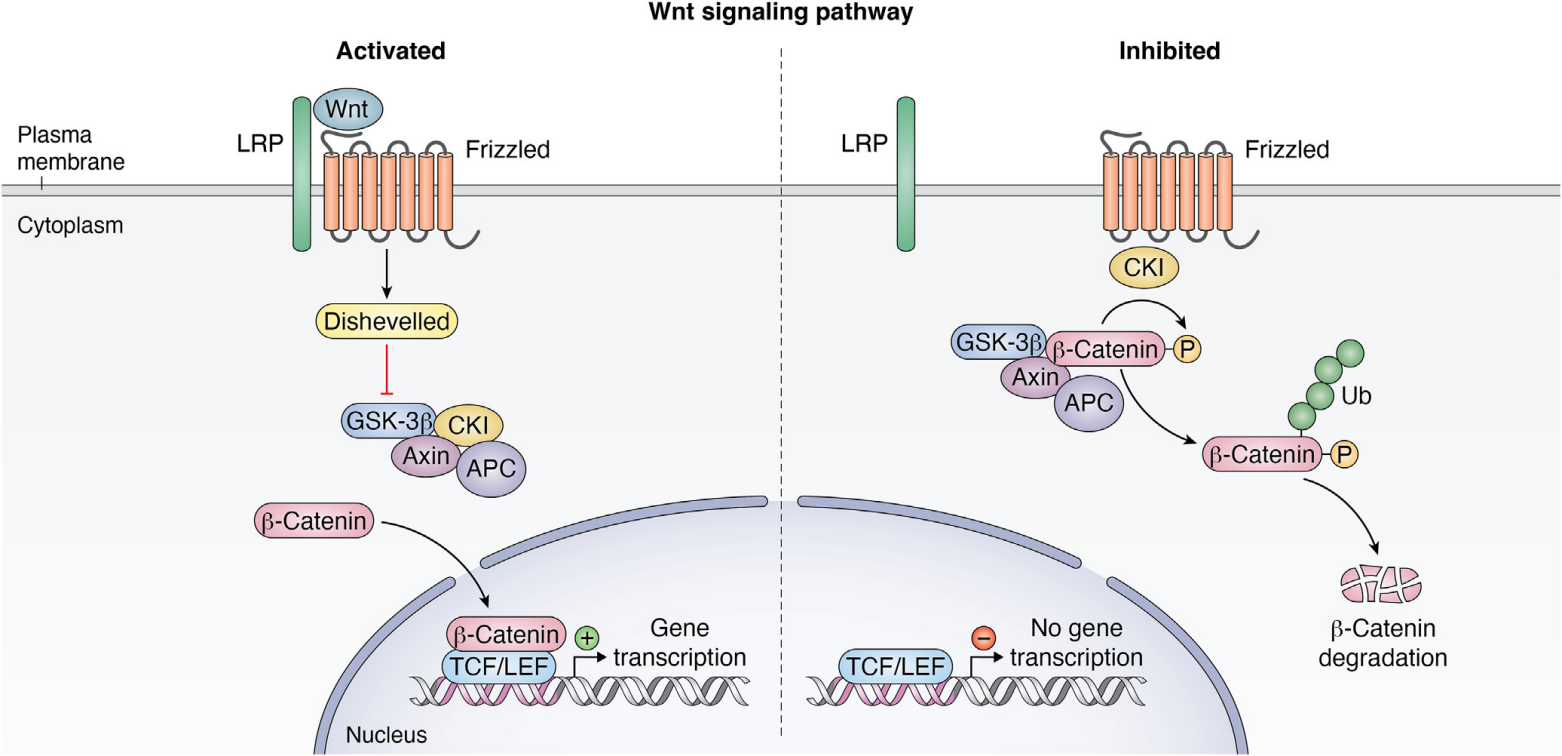
**Wnt/β-Catenin pathway.** The conserved Wnt/β-Catenin pathway regulates stem cell pluripotency and cell fate decisions during development. The Wnt ligand is a secreted glycoprotein that binds to Frizzled receptors, leading to the formation of a larger cell surface complex with LRP5/6. Activation of the Wnt receptor complex triggers displacement of the multifunctional kinase GSK-3β from a regulatory APC/Axin/GSK-3β-complex. In the absence of Wnt-signal (off-state), β-catenin, an integral E-cadherin cell-cell adhesion adaptor protein and transcriptional coregulator, is targeted by coordinated phosphorylation by CK1 and the APC/Axin/GSK-3β-complex leading to its ubiquitination and proteasomal degradation through the β-TrCP/Skp pathway. GSK, glycogen synthase kinase.

When it comes to Wnt canonical signaling, glycogen synthase kinase 3β (GSK3β) phosphorylates β-catenin in the absence of ligands, causing β-catenin degradation and blocking its translocation from the cytoplasm to the nucleus (176) (Fig. 1). In contrast, Wnt3a and Wnt1 ligands combine with LRP coreceptors and FZD receptors when present, and these receptors are phosphorylated by CK1α and GSK3β (177). To enter the nucleus, β-Catenin disassociates from the Axin complex. β-catenin enhances the engagement of histone-modifying coactivators, activating the transcription process (178) (Fig. 1).

The noncanonical Wnt pathway does not involve β-catenin and is also called the Wnt/calcium pathway (176). Dishevelled is activated by Wnt ligands and the ROR-Frizzled receptor. Dishevelled exhibits a peculiar ability to inhibit possible connections with the small GTPase Rho and the protein DAAM1, which, when combined, can trigger Rho kinase and c-Jun N-terminal kinase to promote the rearrangement of the actin cytoskeleton, shaping the cell (179, 180). The noncanonical Wnt/calcium pathway regulates the release of calcium from the endoplasmic reticulum, consequently regulating processes like cell adhesion, migration, and tissue separation through calcineurin and Ca2+/calmodulin-dependent protein kinase II (181).

Some mutations, such as Axin mutations in gastrointestinal malignancies or in GSK3 (which is essential for β-catenin control) do not appear to be associated with cancer formation (182). Certain genes may be able to suppress APC, Axin, and GSK3β activity, increasing β-catenin release into the cytoplasm. These genes include pyruvate kinase isozyme M2 (PKM2) in certain solid tumors, such as breast cancer, telomerase promoter in prostatic cancer, and miRNA in colon and NSCLC (76, 182, 183).

The activation of signaling pathways and transcriptional circuits in stem cells may change the modification or reactivation of these pathways, preventing tumor dormancy, which may lead to tumor growth or cell dissemination (184, 185). Primary cancer dormancy is different from metastatic dormancy, as in primary cancer, cancer cells can either proliferate or be in quiescence to guarantee the molecular stability essential for survival (186, 187). In metastatic dormancy, disseminated tumor cells become dormant depending on the tumor-specific organ microenvironment (4, 187, 188). In these circumstances, cells induce a cytokine cascade, and Wnt maintains the ability for CSCs to self-renew (188). Wnt activation may transform dormant CSCs into activated CSCs, promoting progression through the upregulation of cyclin D1 and Myc. Aberrant Wnt signaling plays a role in the self-renewal of CSCs (189). Recent research has demonstrated that several protooncogenes can activate the Wnt signaling pathway, including PKM2, which is one of four enzyme isoforms and is widely expressed in several tumor types (190). PKM2 significantly impacts the regulation of CSC proliferation in breast cancer. The zeste homolog 2, part of the polycomb PRC2 complex, enhances Wnt/beta catenin hyperactivation, increasing the self-renewal capacity of CSCs (191). Similarly, in gastric cancer, the overexpression of nuclear β-catenin is associated with CSCs self-renewal, consequently increasing tumor aggression. Long noncoding RNAs and miRNAs can induce CSC self-renewal through Wnt activation (192, 193).

Wnt signaling plays other important roles in the dedifferentiation process, including in *de novo* CSC formation. For example, in the peripheral nervous system, PMP22, an integral membrane glycoprotein in myelin, promotes CSC differentiation in gastric tumors; however, mRNA levels decrease due to the Wnt/β-catenin signaling process (194, 195). The prevention of colorectal CSC differentiation *via* β-catenin ubiquitination and phosphorylation triggers the downstream Wnt signaling pathway, mediated by LGR 5. The monitoring of differentiation in esophageal CSCs is another intriguing feature (196, 197).

Apoptosis control by CSCs is another crucial factor. For instance, in CSCs in breast tissue, β-catenin activity is suppressed by apoptosis and G0/G1 arrest (76). DACT1 lowers the amounts of active phospho-β-catenin by promoting apoptosis in breast cancer CSCs (198, 199).

TAZ/yes-associated protein can modulate the phosphorylation status of their targets and, in the absence of inhibitors, can enhance β-catenin activity (200). CDH11 inhibits CSC dissemination in colorectal tumors *via* inhibiting Wnt/β-catenin and AKT/RhoA signaling. Wnt signaling reduces HOXA5 levels, inducing metastasis (201).

#### NOTCH signaling pathway

The Notch pathway is a conserved molecular pathway present in different multicellular organisms. It regulates cellular fate during development and maintains the homeostasis of adult cells (202, 203). The pathway relies on Notch receptors on signal-receiving cells and NOTCH ligands. The Notch family of transmembrane receptors consists of four protein paralogs (NOTCH1–4) (203, 204). There are five NOTCH ligands: the delta-like (DLL) proteins (DLL1, DLL3, and DLL4) and Jagged1 (Jag1) and Jag2 (203, 204). According to Siebel and Lendahl (2017) (205), Notch signaling is a highly conserved intercellular communication pathway that regulates cell specification, survival, and cell differentiation during lung development. While maintaining homeostasis *in vivo* requires normal Notch signaling, abnormal Notch signaling activity has been linked to the initiation and advancement of lung cancer (206, 207). In SCLC, ASCL1 hypomethylation is frequent. DLL3, a NOTCH inhibitor and direct target of ASCL1, showed a strong correlation with its expression level (208).

**Figure 2:**
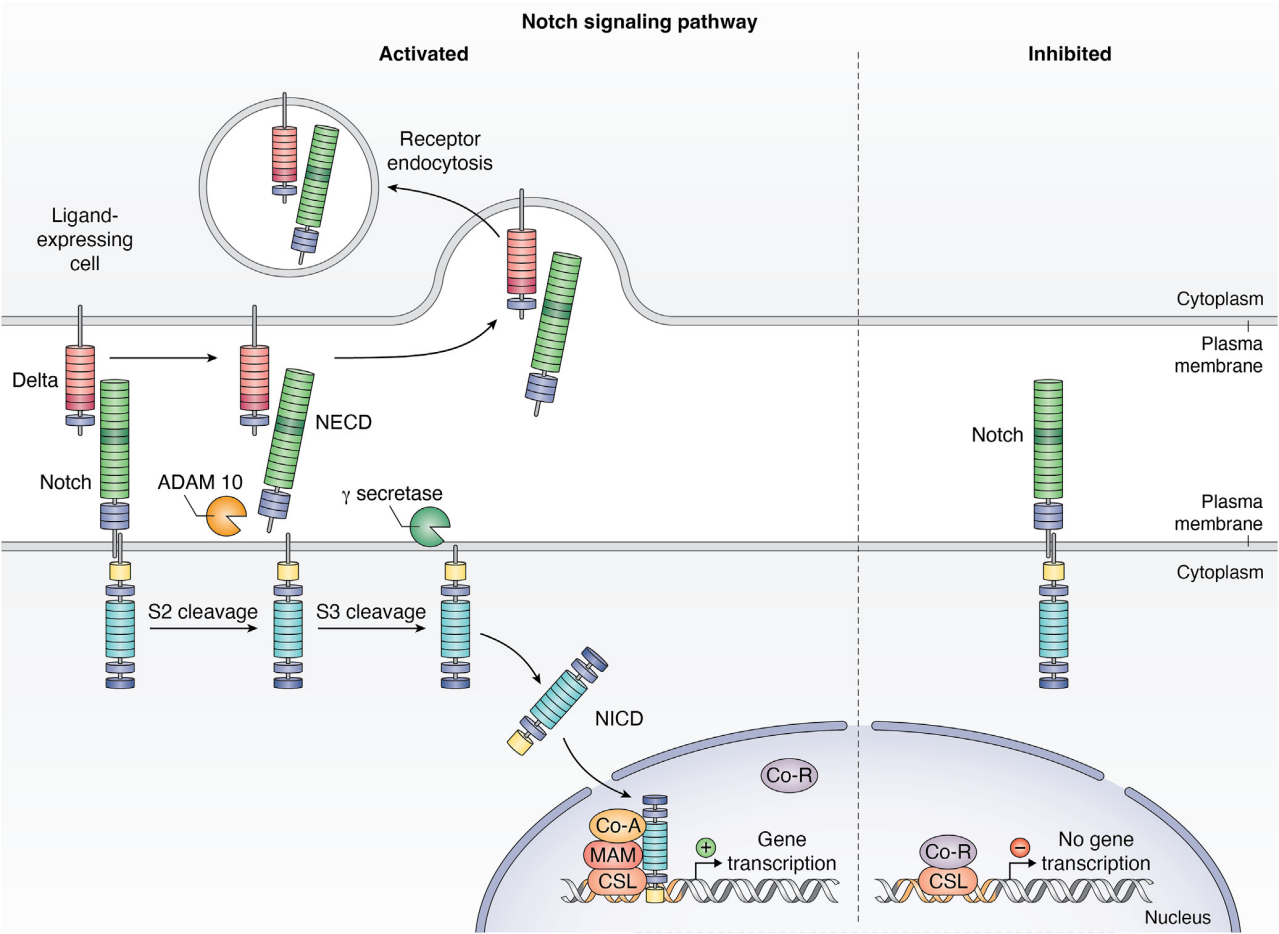
**Canonical Notch signaling pathway.** The unactivated Notch receptor releases a soluble intracellular domain (NICD) after three hydrolysis events. Subsequently, the NICD translocates to the nucleus, where it forms a complex with the DNA-binding protein cellulose synthase-like, displacing a histone deacetylase (HDAc)-corepressor (CoR) complex from CSL. Components of an activation complex, such as MAML1 and histone acetyltransferases (HATs), are recruited to the NICD–CSL complex, leading to the transcriptional activation of Notch target genes.

Under physiological conditions, these ligands bind to NOTCH receptors expressed on nearby juxtracrine cells (208). This results in the removal of the Notch intracellular domain by proteolytic cleavage and the translocation of the transcription factor CBF1, Suppressor of Hairless, Lag-1 which is a transcription factor that activates downstream the Notch signaling pathway (209, 210) (Fig. 2). In particular, the formation of the Notch intracellular domain/CBF1, Suppressor of Hairless, Lag-1 transcriptional activation complex triggers the activation of the target genes, including *HERP, HEY,* and *HES*, basic-helix-loop-helix transcription factors that play crucial roles in tumor growth and progression (211, 212, 213) (Fig. 2).

The Notch pathway regulates cancer cells in several solid tumors, such as breast, colon, and lung cancer, exhibiting specific Notch ligands and receptors (214). Consequently, Notch acts as both an oncogene and suppressive gene in several solid tumors, with the processes appearing to be influenced by the TME. Posttranslational Notch receptor alterations may induce affinities for specific ligands and impact their half-lives (215).

The connection between the Notch and CSCs is based on the activation of the Notch pathway, which impacts cell survival, self-renewal, cancer cell progression, and apoptotic inhibition. Aberrant Notch signaling (*NOTCH1* and *NOTCH4*) confers the capacity for self-renewal and progression to breast and HCC stem cells (76, 216); the CSC state can be triggered as a consequence of the aberrant notch signal (217, 218). The activation of actin-related proteins requires the DLL1-mediated activation of *NOTCH1* to maintain stem cell phenotypes in glioma TICs. Intracellular genes, like *MAP17* (*DD96, PDZKIP1*), a membrane protein located in cells, can regulate the Notch signaling pathway. *MAP17* acts with *NUMB via* a PDZ-binding protein to activate Notch in cervical CSCs (219). Notch signaling activation in liver CSCs has been found to result from the induction of nitric oxide synthase, which promotes the self-renewal process. The enhancement of the CSC-like phenotype in oral squamous cell carcinoma cells by tumor necrosis factor-α (*TNFα*) is due to *NOTCH1* activation (220). *NOTCH1* and *JAG1* are upregulated in breast cancer by *KLF4* and *BMP4*, which, in turn, influences CSC migration and spread. BRCA 1 is a crucial regulator of cell differentiation and is located in an intronic enhancer region within the Jag1 gene, conserving breast CSC stemness (221, 222). The Gli3 protein promotes proliferation and progression in oral CSCs. Hypoxia activates *NOTCH1* and *JAG2*, consequently enhancing self-renewal, invasion, and progression into the metastatic phase (223).

#### Hh signaling pathway

**Figure 3:**
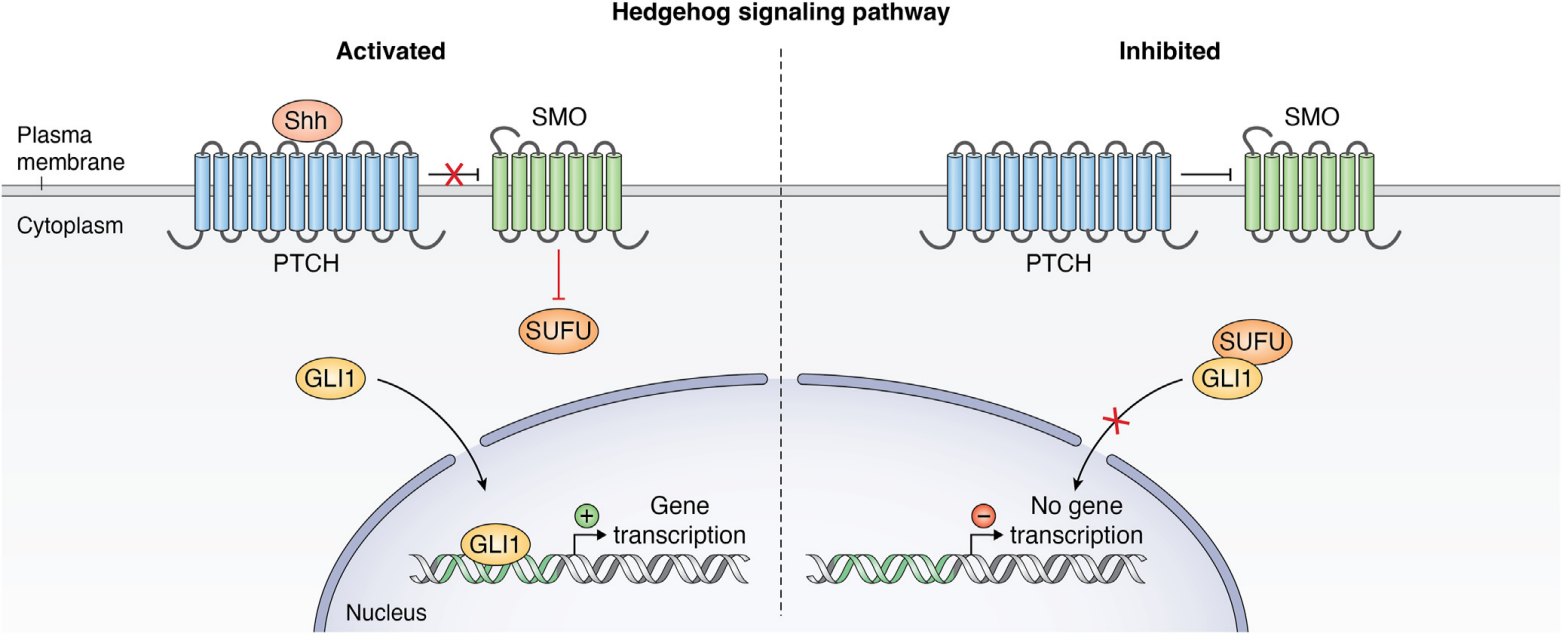
**Hedgehog signaling pathway.** Activation of the HH-GLI signaling pathway occurs *via* the binding of HH ligands to the receptor Patched 1 (PTCH1), which unleashes the activity of the G-protein–coupled transmembrane receptor Smoothened (SMO). In this way, active state of SMO initiates a complex intracellular cascade leading to the activation of the three GLI transcription factors, the final effectors of the HH–GLI pathway. Hh, Hedgehog; SMO, smoothened.

The Hedgehog (Hh) signaling pathway involves a complex network of ligands and receptors. The smoothened (SMO) membrane receptor acts as a positive regulator, while the PTCH membrane receptor has a negative effect (224, 225) (Fig. 3). There are two PTCH subtypes, PTCH1 and PTCH2, with high homology between them. Gli proteins, in particular, GLI1, GLI2, and GLI3, are transcriptional effectors that play key roles in organ and tissue growth (226). These proteins are deregulated in early birth defects and tumors. GLI1 and GLI2 strongly activate transcription, while GLI3 inhibits it. GLI2 is both an activator and inhibitor; however, its main function is as an activator. Several studies have shown the involvement of Hh signaling in embryogenic tissue development (*e.g.*, lung, heart, and skeleton) (227, 228).

Hh ligands bind PTCH receptors on the cell membrane, activating a signaling cascade of intracellular processes by changing the spatial structure of PTCH, and inhibiting SMO binding (229). This impacts Gli, leading to its migration to the nucleus and subsequently regulating cell growth, dissemination, and differentiation. In the other case, without any ligands PTCH receptors bind SMO, preventing the cascade (230, 231) (Fig. 3).

In specifics, aberrant Hh signaling has been linked to a number of solid cancers, such as those of the breast, stomach, and lung (230, 231, 232, 233, 234, 235, 236, 237). Gorlin syndrome is an autosomal disorder linked to *PTCH1* gene loss that includes the basal cell nevus. Many tumors, including medulloblastoma, rhabdomyosarcoma, and basal cell carcinoma, frequently have this gene lost (238).

Hh pathway components may exhibit mutations in *GLI1* and *GLI3*, as seen in pancreatic cancer, or in the *GLI1* amplification gene, as seen in glioblastoma (239). Other genes able to regulate the Hh signaling pathway include the POZ protein, which inhibits Hh signaling and increases Gli2 destruction in gastric tumors (240). Therefore, Hh signaling plays various roles in different tumors. Hh signaling drives tumor development, growth, progression, and therapy resistance (241). In CSCs, Hh signaling is highly active, maintaining cell proliferation, self-renewal, and tumor development capabilities. In glioma stem cells, SMO, GLI, and PTCH promote all CSC activities, including proliferation, self-renewal, and migration (76, 242, 243, 244).

The ERK/PIK3/Hh signaling pathway is one of the regulatory pathways impacted by aberrant DNA methylation of 256 negatively correlated genes. Shi *et al.* (2017) (245) described that this signaling pathway controls both cell survival and death and is linked to the development of squamous cell tumors. Fu *et al.* (2016) (246) showed that *GLI3*'s stability and ability to bind to DNA are enhanced by Set7-mediated methylation at the K436 and K595 sites. This enhances GLI3's stability and contributes to the development of NSCLC by activating Shh signaling.

There are many different ways to alter histones, two of which are acetylation and methylation. Covalent histone modifications have two main functions: they increase transcription and suppress the expression of particular genes. Some histone lysine methyltransferases can methylate the K20 sites in histone H4 and the K4, K9, K27, K36, and K7 sites in histone H3. This is according to Wang *et al.* (2018b). Guo *et al.* (2019) found that while methylation of H3K9, H3K27, and H4K20 inhibits gene transcription, methylation of *H3K4*, *H3K79*, and *H3K36* increases it.

Transcription is made easier by histone acetylation, which reduces the affinity of histones for DNA. On the other hand, transcription is inhibited by histone deacetylation, which eliminates acetyl groups *via* histone deacetylases (HDACs) (247). Research on the triptolide-induced epigenetic modification of H3 histones in NSCLC revealed that methylation of H3 histones, including H3K79me2, reduced the expression of multiple WNT repressors to enhance WNT signaling (248).

KDM5A is a H3K4 demethylase that not only modifies H3K4 methylation levels but also correlates with NOTCH 2 in ASCL1 and neuroendocrine differentiation. This highlights the role that H3K4 methylation plays in the development of small cell lung cancer even more (249). By changing the promoter region's histones' acetylation state, HDACs may have an impact on the development of lung cancer and the Hh pathway. Wei *et al.* (2021) (250) suggests that HDAC may interact with GLI1 to induce the expression of the *SOX2* promoter.

Moreover, cells may be more susceptible to transformation if pretranscriptional DNA methylation combined with histone modifications occurs. Consequently, one key tactic for preventing the growth of lung cancer is comprehending the processes underlying the pretranscriptional epigenetic regulation of stem cells.

## CSCs differentiation, dedifferentiation, and transdifferentiation

The goal of regenerative medicine is to restore the human body's damaged or lost cells, either naturally or by using healthy transplanted cells. While human ES cells were initially considered the best source for cell therapy and human tissue repair, Yamanaka and colleagues' discovery completely changed the field (251). Almost any differentiated cell can be returned to its pluripotent state by expressing the appropriate transcription factors. An understanding of the potential ancestry of CSCs can be gained through somatic reprogramming using Yamanaka factors, many of which are oncogenes (251). It is crucial to comprehend these processes since doing so may enhance knowledge and make it necessary to think of fresh approaches and new strategies to fight against cancer (251).

Specifically, CSCs can differentiate into endothelial cells, pericytes, cancer-associated fibroblasts (CAFs), stromal cells (including mesenchymal cells), or cancer cells. Nevertheless, it’s critical to comprehend every aspect of the regeneration process, including the ability of differentiated cells to dedifferentiate into progenitor cells, the ability of cells to transdifferentiate into different cell types, and the ability of somatic cells to be stimulated to become pluripotent cells (reprogramming). In particular, *differentiation* is a constant process during their cell life, maintaining homeostasis; *dedifferentiation* is the process by which cells move from a partially or terminally differentiated stage to a less differentiated stage (4, 252); *transdifferentiation* is the process by which a specialized and mature cell type changes into another without going through a pluripotent state. It is also known as lineage reprogramming or conversion, by expressing transcription factors and/or other stimuli (253, 254).

There is still a great deal to learn about organ and tissue regeneration and restoration. Researchers have discovered that certain somatic stem cells, such as those found in liver or blood tissues, can be activated to regenerate tissue, as can the induction of differentiated cells capable of proliferation (255, 256, 257). Some vertebrate species have demonstrated the ability to replace parts of the body, such as limbs. By studying these regenerative processes, scientists could uncover cellular and molecular mechanisms that could help create regenerative strategies for humans (258, 259).

One of the most important mechanisms that could aid these strategies is the dedifferentiation process. This process induces cells to proliferate before redifferentiation, meaning lost cells could be reproduced (260, 261). Transdifferentiation is a natural mechanism that was first observed by scientists over 100 years ago, through which cells can regress into lineages and differentiate into other cell types (262). Although the reprogramming step could be considered a genuine regenerative process, it has not yet been formally declared as such (263). Furthermore, the reprogramming processes remain poorly defined due to extreme interpatient variability, especially due to immunological differences (264, 265, 266, 267).

Another interesting example of dedifferentiation is limb regeneration in amphibians. After amputation, cells start to dedifferentiate, creating a heterogeneous cell mass that, through migration and proliferation, transiently forms at the injury site and undergoes morphogenesis, forming the missing limb (268). This process involves undifferentiated cells which proliferate and redifferentiate (265). The process of dedifferentiation has been widely studied for both its role in benign regenerative medicine and in tumor regeneration. Tumor re-entry into the cell cycle is crucial for cell differentiation in retinoblastomas (269, 270). The main hypothesis suggests that cells dedifferentiate, entering the cell cycle and reverting to pluripotent or stem cells; however, it has been suggested that dedifferentiated blastoma cells persist in their original lineage (271). The nonregression of cells to pluripotency is an interesting consideration. When regression does occur, cells appear to retain memory, likely epigenetic, of their tissue of origin (272).

The complexity of the dedifferentiation process is linked with the cell cycle, although the dedifferentiation process itself does not appear directly associated (261). The *drosophila melanogaster* model is significant for examining the functions of the tumor suppressor retinoblastoma protein (273). The dedifferentiation process continues even when proliferation is blocked. This implies that retinoblastoma protein contributes to maintaining cell differentiation and that dedifferentiation and the cell cycle are two different processes (262, 274). For example, Schwann cell dedifferentiation seems to be uncoupled from cell proliferation. Furthermore, cAMP removal induces cell dedifferentiation without inducing proliferation. Cells may be stimulated to differentiate and dedifferentiate without entering the cell cycle (275).

The conversion of damaged tissues into differentiated cells of a consistent cell type is another interesting process in terms of regeneration (276). This mechanism involves two steps: 1) cell dedifferentiation and 2) natural cell differentiation. Transdifferentiation enables a tissue to regenerate into a different tissue type (277). To achieve this, cells first need to dedifferentiate (278). Then, they naturally proliferate, creating new cells that differentiate into mature cells of the “new” tissue (225). Blocking the cell cycle is not enough to stop the transdifferentiation process or the consequent formation of new cells. Therefore, the primary prerequisite for initiating the transdifferentiation process is the dedifferentiation process (254, 279).

### Dedifferentiation in CSCs

The process by which a differentiated cell returns to a precursor cell type belonging to the same lineage is known as dedifferentiation (280). Stem cell–like phenotypes have been linked to ectopic transcription factors, such as SNAIL and TWIST1 (281). Another study reported that intestinal epithelial cells can activate RAS and nuclear factor kappa B (NF-κB) to promote tumor growth by transitioning to states that resemble stem cells (282). These findings suggest that the dedifferentiation-induced conversion of cells into CSCs may be promoted by genetic and epigenetic mutations. Therefore, dedifferentiation can produce more CSCs (283).

Bonnet and Dick (7) noted that leukemic cells could be differentiated from CD34+/CD38− cells isolated from patients with AML (284, 285). Numerous studies have shown that cells from various cancers, such as pancreatic, lung, and liver cancers, can dedifferentiate (286, 287). Additionally, CSC dedifferentiation has been noted in sarcomas. Multiple differentiation lineages observed in mesenchymal stem cells (MSCs) have been proposed to be driven by transformed mesenchymal cells (288). Numerous malignancies have been found to induce dedifferentiation when exposed to CAFs. According to Chen *et al.* (2014), lung CSCs in lung cancer developed a “niche” for CAFs, and when those CAFs were removed, the lung CSCs dedifferentiated (289).

### Transdifferentiation in CSCs

Stromal cells and other TME cells, such as TME immune cells, are often recruited by tumor cells to contribute to shaping a microenvironment more supportive of tumor cell growth (290). To survive and proliferate, cancer cells must have specific mechanisms, which they acquire by transdifferentiating into various stromal cell types (291, 292). Transdifferentiation, also called lineage reprogramming, involves the phenotypic change from a pluripotent state to another mature cell type without going through a transition (293). CSCs can differentiate, just like normal stem cells. However, unlike normal stem cells, they can also transdifferentiate into different types of endothelial cells (294, 295, 296).

Transdifferentiation has been described in a variety of cell types within the TME. For instance, vascular mimicry in melanomas has been documented, in which certain cells simultaneously express markers for both malignant and normal endothelial cells (288). Vascular mimicry is the process by which tumors, independent of the usual methods of angiogenesis, create their own tumor cell–lined channels for fluid transport. Vascular mimicry has been demonstrated in several cancers, including breast, ovarian, and lung cancers. CD105+ renal CSCs have demonstrated the ability to produce CSCs and stimulate angiogenesis both *in vitro* and *in vivo* (297). It has also been showed that mammary gland cancer cells can differentiate into endothelial cells in both *in vitro* and *in vivo* models (297).

Alvero *et al.* (298) observed comparable outcomes in *in vivo* models of ovarian and breast CSCs, validating the function of CSCs as vascular progenitors. In addition, it has been noted that glioblastoma CSCs transdifferentiate into mural cells, specifically pericytes, to preserve the tumor vasculature (299, 300). Most stromal cells in this niche were CAFs, and transdifferentiation has been documented in these cells. CAFs can originate from a variety of cell types, including stem cells obtained from adipose tissue, smooth muscle cells, myofibroblasts, mesenchymal cells, and normal resident fibroblast cells (301).

The genesis of CAFs is influenced by transitions from epithelial to mesenchymal and endothelial to mesenchymal tissue (302). MSC progeny have also been noted to exhibit transdifferentiation. Bone marrow–derived MSCs, which are found in the TME, have a distinct life trajectory that enables them to differentiate or transdifferentiate into a variety of cell types, such as activated fibroblasts, pericytes, and macrophage-like cells, all of which promote tumor growth (303).

### Fusion of cells

The process of merging two cells’ plasma membranes to create a single heterokaryon or synkaryon cell is known as cell-cell fusion. This mechanism significantly impacts several physiological functions, including placentation, fertilization, osteoclast genesis, and tissue regeneration, as well as pathological diseases, such as infections and cancer (304, 305). The fusion process involves three steps: 1) a prefusion stage during which a nonfusogenic cellular state is transformed into a profusogenic one *via* the activation of fusogenic machinery (chemokines, adhesion molecules, and fusogens), altering composition of the plasma membrane; 2) fusion, which involves the fusion of two plasma membranes aligned at a specific distance mediated by fusogens, such *as SYNCYTIN-1, SYNCYTIN-2, EFF-1, IZUMOL, AFF-1, CD9, JUNO, MYOMAKER,* and *MYOMERGER*; and 3) the postfusion phase, which stops additional unchecked cell-to-cell fusion events by shifting the profusogenic cellular state to a nonfusogenic one (285, 306).

Cell fusion can be heterotypic or homotypic. In the former, cells from different lineages coexpress genes from both cells, resulting in a blended phenotype (307). In the latter, the fusion of cells from the same lineage and differentiated state results in bigger daughter cells that possess stronger features and usually more robust functions (308, 309). To create cancer hybrid cells, cancer cells can merge with other cancer cells as well as with healthy cells, such as fibroblasts, macrophages, stromal cells, and stem cells. The process of dedifferentiation and cancer initiation has been linked to the fusion of myeloid cells with tumor cells, which has been shown to improve tumor proliferation and survival, invasion and metastasis, therapy resistance, and immune evasion (285, 310). Tumor suppressor genes can be transmitted to daughter cells, offering the potential to revert to a neoplastic state when cancer cells are fused with normal ones (311, 312, 313).

Cancer hybrid cells have been reported in ovarian, breast, lung, and gastrointestinal tract cancers. It has been suggested that the heterotypic fusion of stem cells and cancer cells results in the production of daughter cells exhibiting characteristics similar to CSCs (4, 306, 314). According to Wei *et al.* (315), the hybrid cells created when lung cancer cells and MSCs fuse express stem cell markers, like OCT4, SOX2, NANOG, and KLF4, and demonstrate enhanced metastatic potential and epithelial-to-mesenchymal transition (EMT).

When human umbilical cord MSCs and gastric cancer cells merge *in vitro*, tumorigenic hybrids are created that demonstrate increased expression of stemness markers, such as CD44, CD133, OCT4, SOX2, and NANOG (316). The fusion of breast cancer cells with mesenchymal stem/stromal cells primarily produced CD44+/CD24−/low hybrid cells with variable ALDH positivity (317). The combination of breast epithelial and human breast cancer cells with a stem cell phenotype has been shown to generate daughter cells with higher ALDH positivity that express SOX9 and SLUG (318).

The tumorigenicity, proliferation, colony-forming capacity, metastatic potential, and drug resistance of hybrid cells with a CSC-like phenotype are increased. In addition, these cells facilitate tumor growth by evading immune responses, differentiating into different cell types, expanding tumor volume, and generating an environment favorable for tumor survival (4, 37, 319).

### The transitional phases

Cells take some time to acquire pluripotency after priming. Cells can regress back to pluripotency following several events, including stem gene activation, differentiation gene suppression, and epigenetic changes (320, 321). However, whether this is a random process in which different events happen in different cells at different times and in different combinations remains unclear. If so, every cell could potentially return to pluripotency *via* an entirely different path. An alternative theory is that a cell needs to go through a specific series of stages to become pluripotent (320, 321, 322, 323).

To be more precise, stable partially reprogrammed cells only reactivate a small subset of genes associated with stem cells and fail to repress many genes linked to differentiation. Although such cells have not reached pluripotency, further manipulation may encourage them to finish the process stage (321, 322, 323). Imaging individual fibroblasts during the reprogramming process has provided additional evidence regarding the existence of intermediate stages. It has been demonstrated that shortly after induction, a unique class of tiny, quickly dividing cells appear, from which pluripotent colonies develop (324). If this is another common reprogramming intermediate stage, it should be possible to identify it in similar analyses of various cell types in conjunction with genetic and epigenetic data (325). These results could explain these intermediate stages and their role in reaching pluripotency (325, 326).

### Processes comparisons

#### Dedifferentiation *versus* reprogramming

On the surface, reprogramming and dedifferentiation are quite similar, as they both result in the regression of a differentiated cell (262, 325). Reprogramming could be considered the most extreme type of dedifferentiation since it causes the cells to revert to their pluripotent state. Since the primary similarity between these two processes is the regression of a differentiated cell, some researchers question whether reprogramming might also involve other dedifferentiation-related mechanisms (326).

For instance, it appears that a cell must first be “unlocked” from its terminal differentiated state to regress or enter the cell cycle (262, 325). An initial, unidentified event during reprogramming makes it possible for exogenous factors to induce pluripotency. It is unclear whether this is similar to losing terminal differentiation or complete dedifferentiation. Although this unknown process is connected to proliferation, dedifferentiation is a prerequisite for many cells to enter the cell cycle (295). This implies that by taking advantage of the natural process, SOX2 and OCT4 may be able to access previously restricted chromatin, causing further cell regression (327).

This theory is motivated by the fact that mature B cells cannot be reprogrammed until they have undergone dedifferentiation with either *CEBP**A*** or *PAX5*. It is unclear where a dedifferentiated cell can be reprogrammed if the cell cycle is disrupted. Distinct natural intermediate phases linked to dedifferentiation, which resemble a reversal of natural intermediates, have been discovered in the reprogramming process, suggesting that a comparable reversal is unlikely (328).

#### Transdifferentiation *versus* reprogramming

**Figure 4:**
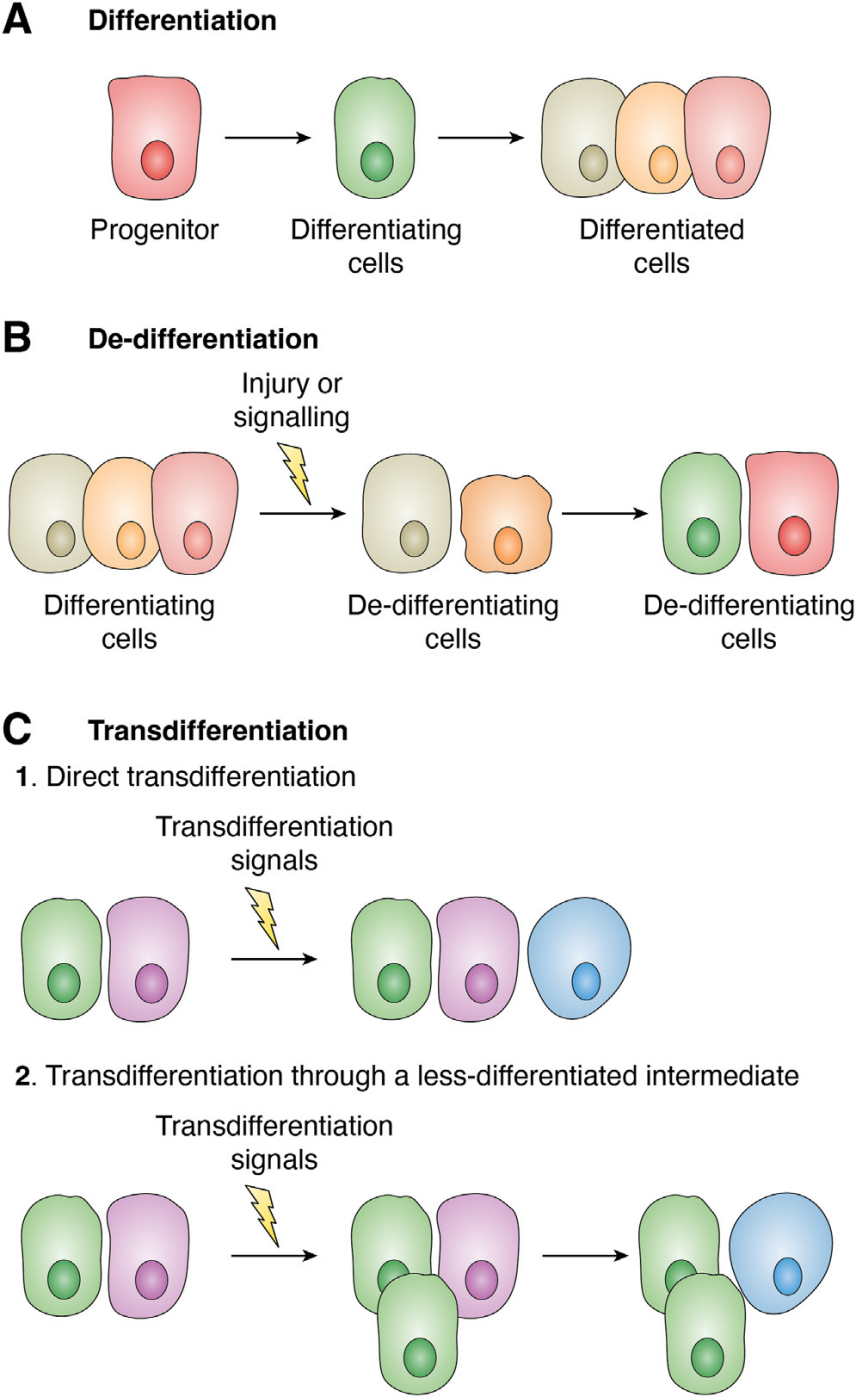
**Differentiation, dedifferentiation and transdifferentiation.***A*, progenitors and stem cells differentiate to form mature, differentiated cells. *B*, in the event of damage also sustained by the stem cells, the differentiated cells may dedifferentiate. *C1* and *C2*, in the case of injury, differentiated cells may also assume a different cell fate of, a process known as *trans-*differentiation which can occur directly, without any immature phenotypes, or through a dedifferentiation phase before the cells redifferentiate to a new mature phenotype.

Transdifferentiation and dedifferentiation have many similarities. One of the transdifferentiation models requires a preliminary dedifferentiation phase (307) (Fig. 4). The differentiation of induced pluripotent stem (iPS) cells into separate lineages appears to demonstrate comparability between reprogramming and natural transdifferentiation, with a dedifferentiation stage that restores the cell to pluripotency before redifferentiating into a new lineage (329).

Transdifferentiation between closely related cells has been proposed to occur when one program is downregulated and another is activated simultaneously, resulting in an aberrant intermediate phenotype. Examples of these related cells include B cells and macrophages or α cells and β cells (330, 331). In addition to pluripotency, reprogramming could result in the emergence of artificial intermediates in cells that express genes exclusive to a certain lineage (88). This is similar to the aberrant intermediates seen during the transdifferentiation of B cells into macrophages and α cells into β cells (330). These intermediates are prone to instability and likely represent the pace at which this process proceeds after mRNAs and proteins take over (330). However, transdifferentiation occurs between different lineages and produces differentiated cells rather than pluripotent cells, which is what happens during reprogramming. Therefore, there is not enough information available to determine whether transdifferentiation and reprogramming are truly similar (254, 332).

#### Applicability

Reprogramming, transdifferentiation, and dedifferentiation may be used as therapeutic approaches in regenerative medicine (262). Regarding their present *in vivo* potential, the three processes differ significantly. Although dedifferentiation and transdifferentiation can be accomplished successfully *in vivo*, the process of guiding pluripotent cells into a new lineage has only been accomplished *in vitro* (333).

The ability to generate new cells *in vivo* removes the need for cell transplantation and its associated problems. Reprogramming provides the possibility of enabling genetic cell modification. This has numerous implications, one of which is the repair of disease-causing mutations (334). It is possible to create clonal populations of genetically modified iPS cells *in vitro*, guaranteeing that only appropriately altered cells are chosen for further application. Moreover, these populations can be grown, which is particularly relevant if the goal is to replace cells that have been lost due to illness or damage. All three processes have benefits for regenerative medicine, despite causing major changes in differentiated cells (335).

Patients may be able to regain cells lost to illness or injury by having their cells reprogrammed *in vitro*, differentiated into the right cell type, and then grafted back *in vivo*. Inducing less specialized or more common cells to transdifferentiate into target cell types would be a simpler approach than inducing cells to dedifferentiate and then proliferate (320). Transdifferentiating or dedifferentiating the cells will not help if the aim is to correct a genetic mutation that causes the illness, as the new cells will still carry the mutation. Reprogramming a patient’s cells *in vitro*, fixing the mutated gene, differentiating the cells into the right lineage, and then transferring the cells back would be one course of action (254, 262).

The concepts of dedifferentiation, transdifferentiation, and reprogramming have altered the current understanding of differentiated cells, and their potential uses in regenerative medicine (209). Cells can use each of these processes to drastically alter their characteristics. In particular, reprogramming has sparked renewed interest in the transdifferentiation field. Transdifferentiation is effectively reprogramming that uses pooled transcription factors followed by serial subtraction. However, reprogramming differs greatly from dedifferentiation or transdifferentiation in that it appears to be a very artificial process (262).

It may be beneficial to modify reprogramming techniques to address pluripotency in a more organic manner (325). For instance, to enable a smoother and more efficient shift from differentiated cells to pluripotency, it would make sense to begin the reprogramming process with a step or steps of dedifferentiation. Research can concentrate on the final stages of the induction process and potentially reduce reprogramming stochasticity by employing cells with specific lineages and dedifferentiation potentials (336). Furthermore, if a certain cell type is to be reprogrammed, transdifferentiation, which involves transcription factors exclusive to a given lineage, could also cause cell regression. Currently, one of the biggest issues with reprogramming is its safety (337). The stress that reprogramming places on cells may cause iPS cells with altered stress-regulating genes to be selected, making them more prone to tumor growth (338).

Helping a cell regain pluripotency may be better achieved gradually, as in the case of natural dedifferentiation and transdifferentiation. Not only will a deeper comprehension of these mechanisms facilitate the therapeutic application of each process in isolation but it may also be applied universally to broaden the collective understanding of the three processes (339).

## CSCs plasticity

**Figure 5:**
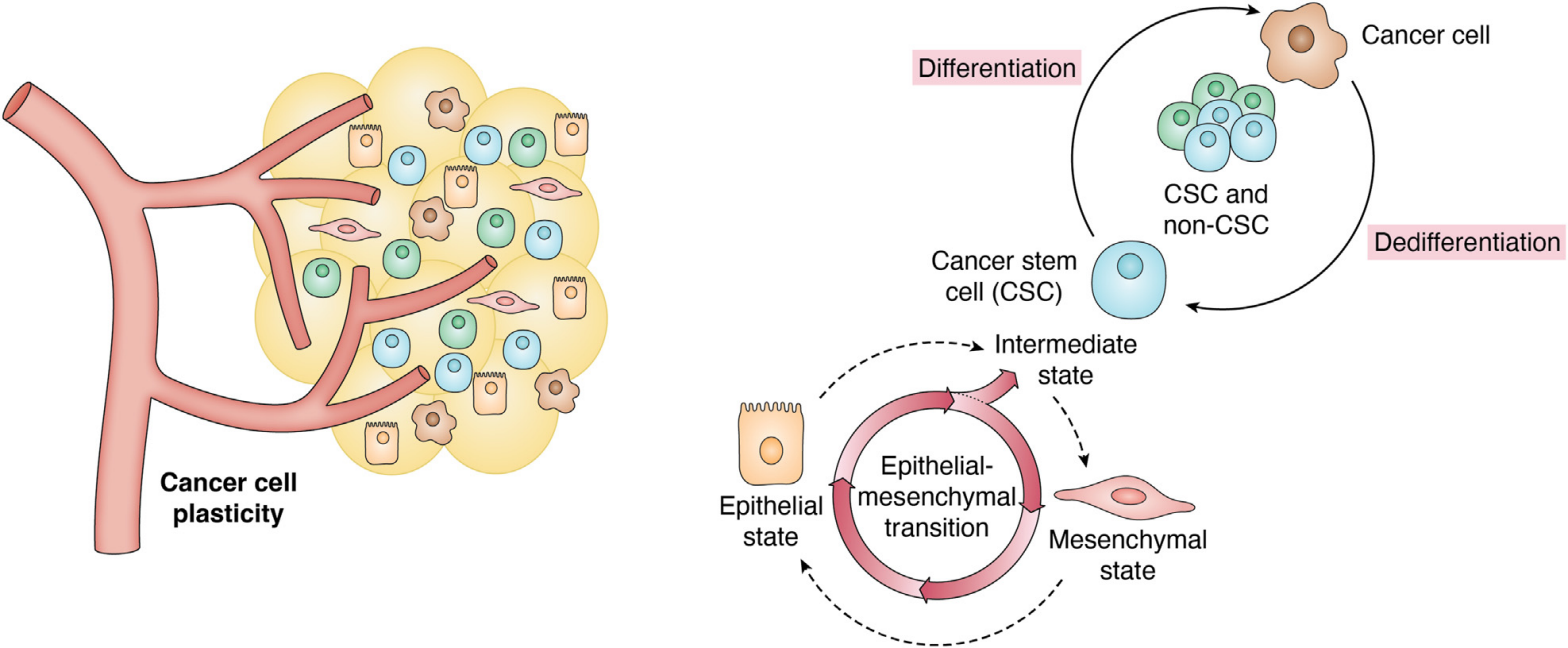
**CSC plasticity.** Cancer stem cell plasticity is the ability to dynamically switch between CSC and non-CSC states. Plasticity enables CSCs dynamic and able to undergo spontaneous state transitions, such as the epithelial-to-mesenchymal transition (EMT), which gave rise to the concept that non-CSCs can also convert into CSCs. As a consequence of this cross-conversion of cell populations in a tumour, drug-resistant, and/or metastatic cells increase, resulting ultimately in cancer-associated mortality.

CSCs are cancer cells found inside tumors that have many traits in common with stem cells, such as the capacity to self-renew, develop, and generate various cell types often found in malignancies. Because they are carcinogenic, CSCs cause cancerous growth through the processes of self-renewal and differentiation, which prolongs the disease (58, 340, 341) (Fig. 5). Tannock *et al.* (2013) stated that the standard CSC model implies that each cancer is arranged in a hierarchy, with only a subset of malignant cells able to replicate the morphological and antigenic heterogeneity of the original tumor through self-renewal, proliferation, and differentiation (342). Through the disruption of genes and signaling pathways that regulate the normal process of self-renewal, these cells exhibit dysregulated self-renewal mechanisms that enable tumor cells to multiply without losing their ability to proliferate (343).

CSCs exhibit resistance to cell death pathways through expressing multidrug–resistant ABC transporter proteins, acquiring genetic alterations, and dysregulating proapoptotic and antiapoptotic signals (344). In addition, CSCs demonstrate increased angiogenesis, metastasis, immune suppression, and metabolic reprogramming, which facilitate their endurance, expansion, and penetration, culminating in resistance to therapy and recurrence (345).

The observed plasticity of CSCs is believed to be one of the primary factors driving their characteristics (346). Several studies have documented this plasticity. The chromatin remodeler JARID1b, which is dynamically regulated, was used by Roesch *et al.* (347) to isolate melanoma cells that resembled CSCs. The cells were found to re-express the maker following JARID1b gene knockdown, which defied the hierarchical CSCs model (347).

An unanticipated level of plasticity between non-stem-like and stem-like cells has been documented in human mammary epithelial cells in both *in vitro* and *in vivo* experiments. Without undergoing genetic modification, the plasticity in differentiated mammary epithelial cells was found to be regulated by zinc finger E-box–binding homeobox 1 (ZEB1) transcription, a crucial regulator of EMT (348). The interconversion has been reported to hold therapeutic significance (349).

Colorectal LGR5+ and LGR5 CSCs demonstrated interconversion with and without anticancer drugs. Since both states could recapitulate primary tumors, this genetic ablation has no effect on tumor regression. However, at the secondary or metastatic site, the response differs, indicating that the microenvironment at the primary and secondary sites differentially regulates the processes (350, 351, 352). CSCs have a survival advantage as they can transition between non-CSC and CSC phenotypes due to various types of plasticity (285) (Fig. 5). The transition of a normal cell into a cancerous cell is driven by changes in metabolism, transcriptomics, genetics, and epigenetics. Intratumor and intertumor heterogeneity result from this transition, complicating the development of successful treatments (353, 354). Patient-specific, germline, and somatic mutations cause intertumor heterogeneity, which is seen in patients with the histological subtype of tumor. Intratumor heterogeneity is associated with the uneven distribution of genetically variant tumors within and across tumors (spatial heterogeneity) or dynamic variations in the genetic diversity of a distinct tumor over time (temporal heterogeneity) and is caused by somatic coding and noncoding alterations or modifications at the epigenetic, transcriptomic, and proteomic levels (355, 356).

Three hypotheses have been proposed to explain tumor heterogeneity: the plasticity model (Fig. 5), the CSC model, and the clonal evolution (CE) model. Plaks *et al.* (35) state that the first two models attempt to explain aspects of tumor maintenance, progression, and genesis. These models do not conflict with one another, despite suggesting distinct mechanisms (357). The plasticity model is a combined theory that includes the other two models. The CE model postulates that cancer cells are initially homogeneous with very little variation (358).

Due to their instability, cancer cells are susceptible to epigenetic and genetic modifications, which can influence aspects such as treatment resistance and invasiveness (359, 360). Tumor heterogeneity results from the adoption of characteristics that encourage tumor progression. The inability to distinguish tumor-inducing activities based on the inherent traits of a particular subgroup of cancer cells is a result of the CE model (358).

According to the CSC theory, a tiny percentage of tumor cells may be able to self-renew and differentiate (361). Like normal stem cells, tumorigenic CSCs form a hierarchy, resulting in progenitor cells that differentiate and accelerate tumor growth. Variations in these cells lead to differentiation into different cell types, which are known as cell-type heterogeneity (362). CSCs can multiply quickly because they have the characteristics of cancer cells and are not constrained by traditional growth-inhibiting mechanisms (363). The CSC model, in contrast to the CE model, proposes that intrinsic factors have the capacity to differentiate and isolate CSCs from the tumor (295).

Reversible cell plasticity is characterized by the capacity to change between varying states, such as differentiable and stem cells, asymmetrically and symmetrically dividing cells, quiescent and proliferative cells, and epithelial and mesenchymal cells, as well as drug-sensitive and drug-resistant phases (364). This plasticity is frequently associated with the aggressive cell behavior of CD34+ cells, which aids in cancer maintenance and advancement. Both intrinsic and inherited factors, which act through the activation of different transcription factors responsible for genetic and epigenetic mechanisms, and extrinsic or acquired factors, which are initiated by the TME or niche, can trigger the process (364). According to Hanahan (2), cancer cells can unlock the normally restricted property of plasticity to evade a terminally differentiated state, which is an emerging hallmark of cancer.

Tumor cell plasticity (TCP) during tumor formation is used to explain metastasis and treatment resistance. Examples of this are characterized as the dedifferentiation of tumor cells from normal cells to progenitor-like cell states, which prevents neoplastic cells from properly differentiating from progenitor cells, maintaining partially differentiated progenitor cell states while transdifferentiating to different lineages (2). The different types of plasticity are listed in this review to highlight the significance of cellular plasticity in CSC maintenance, metastasis, and proliferation. Additionally, the role of plasticity in therapeutic resistance and potential approaches to take advantage of this phenotype are covered.

Furthermore, it has been shown that lung CSCs express *SOX2* and plays a crucial role in lung CSC maintenance (365). The findings of this study indicate that *SOX2* may be a key player in the dedifferentiation of lung cancer cells, as demonstrated by the higher levels of upregulated SOX2 expression seen in dedifferentiated CSCs derived from non-CSCs. Specifically, during the dedifferentiation of colon and breast cancer, respectively, Wnt/β-Catenin signaling and the EMT are triggered (366, 367, 368). Thus, dedifferentiation in various cancer types is mediated by distinct molecular mechanisms.

The transcriptional activity of *SOX2* is mediated in ES cells by *POU5F1* (*OCT3/4*), a partner complex of *SOX2* (369). The SOX2 transcription factors in cancer cells are still unknown. Using a luciferase assay, it was determined that *HOXA5* is one of the transcription factors that induces *SOX2* expression. Under HOXA5 overexpression, trichostatin A treatment boosted *SOX2* expression.

Therefore, the state of the *SOX2* promoter's histone acetylation may also have an impact on *SOX2* expression. Nevertheless, HOXA5 has been linked to lung development (370) and is a tumor-suppressor gene that can activate the TP53 tumor-suppressor gene (370). Prior research has demonstrated that in breast and lung cancer cells, DNA methylation renders the promoter region of HOXA5 inactive (371, 372).

Recent findings, however, demonstrated that although 5aza treatment induced *HOAX5* transcription in breast cancer cells, it did not do so in lung cancer cells. These findings suggest that the *HOAX5* promoter region's methylation status is variable and may be influenced by a number of factors.

It has been discovered that acetylation of H3, which can be brought on by oxidative stress-induced suppression of *HDAC8* expression, regulates the *HOXA5* promoter. Thus, by means of histone acetylation and *HOXA5* induction, which is succeeded by *SOX2* expression and *TP53* repression, oxidative stress may contribute to the induction of lung CSCs. Oxidative stress is a constant in the lungs, and in the lung AC cell line A549, it has been demonstrated that oxidative stress induces histone acetylation by repressing HDAC2 (373, 374). These findings suggest that oxidative stress can control the regulation of epigenetic gene expression by suppressing HDACs, though the exact molecular mechanisms remain unknown.

### EMT-induced plasticity

**Figure 6:**
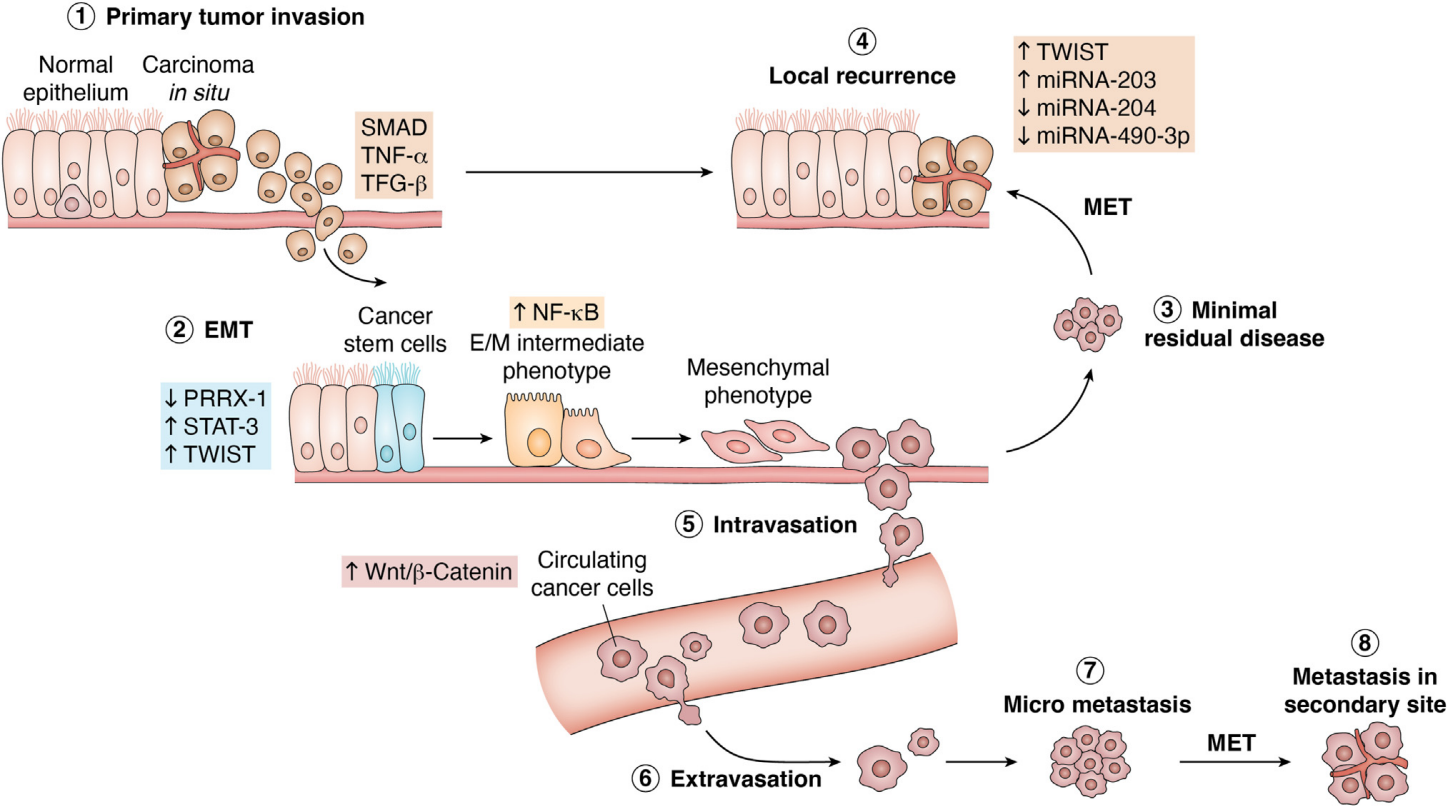
**The role of EMT in tumor progression.** The tumour microenvironment and extracellular stimuli play crucial roles in EMT transformation and tumor progression with a multiplicity of factors like as cytokines and epigenetic factors like miRNAs. EMT, epithelial-to-mesenchymal transition.

The process known as EMT is responsible for the malignant development of cancers. In EMT, the expression of mesenchymal cell markers is elevated, while that of epithelial cell markers are downregulated (375). The cells gain a variety of traits after changing from a monolayer to a mesenchymal state, such as invasiveness, apoptotic resistance, immune evasion, and agility (376, 377, 378). The process known as mesenchymal-to-epithelial transition (MET), in which mesenchymal cells change from one phenotype to another, enables the cells to repopulate tumors from a distance after invasion (379) (Fig. 6).

This phenotypic shift is characterized by multidrug resistance and tumor recurrence (380, 381). EMT and MET can occur in different degrees within cells, indicating that the transition process goes through several stages, encompassing the entire EMT process (380). Consequently, the process of phenotypic switching can be regarded as a plastic program in which cells are able to efficiently transition between mesenchymal and epithelial states (380, 381). Being able to identify the limit of EMT/MET activation within a single cell or within a tumor is extremely important, as it is indicative of tumor growth and metastasis (382). The interaction between cancer and EMT seems to be a possible important target for further consideration. In particular, in a recent study published in 2024 by Yoon Kyung Jeon *et al.* (383) in NSCLC, they investigated how the EMT is influenced by tumor cell-intrinsic PD-L1 signaling and how EMT serves as a predictor target for immune checkpoint inhibitors (ICIs), demonstrating that through the action of TGFβ, cell-intrinsic PD-L1 signaling causes EMT in NSCLC, which in turn accelerates the growth and metastasis of the tumor both *in vitro* and *in vivo*. This study has demonstrated that EMT may serve as predictor for ICI therapy in patients with PD-L1-high NSCLC. It is the first to investigate the relationship between PD-L1 expression and the ICI response in the context of EMT.

A prior study found that gefitinib treatment altered the EMT status of lung ACs (383, 384, 385). More research supported the idea that the Notch-1 signaling pathway was activated during the EMT process, which resulted in the development of gefitinib-acquired resistance. A key player in the neuroendocrine transdifferentiation process of NSCLC is notch signaling. This process allows NSCLC to become SCLC and develop resistance to EGFR-TKIs following initial targeted treatment (384, 385).

Consequently, it has been discovered that the Notch pathway is necessary for prostate cancer cells' EMT process, facilitates a change toward basal stem-like characteristics, and results in a phenotype that is resistant to castration. Tumor invasive regions exhibit significant expression of Notch signaling pathway components, indicating the pathway's critical role in the regulation of EMT. A recent study by Natsuizaka *et al.* (386) suggests that Notch1 signaling and EMT work together to promote the growth of tumors that cause squamous cell carcinoma.

Furthermore, TGF-β, other significant protein, directs *NOTCH1* to promote EMT (387). According to a study by Zeng *et al.* (387), blocking *NOTCH1* causes breast cancer cells to become resistant to cisplatin and reverses the EMT process.

Additionally, the data revealed that Notch1 overexpression is associated with a worse prognosis for triple-negative breast cancer (TNBC) patients. The regulation of EMT transcription factors is linked to the NOTCH-mediated activation of EMT, as shown by Saad *et al.* (388).

Snail and Slug are two transcription factors that contribute to the Notch pathway–mediated inhibition of β-catenin and E-cadherin (389). The suppression of Notch activation reduces Snail expression while restricting the rise in MMP-2 and MMP-99 expression. Direct evidence that NOTCH signaling causes EMT through the Snail pathway was presented by the study. The EMT process can be facilitated by Notch signaling working in concert with other pathways (389).

Partial EMT is the term used to describe the situation where epithelial carcinoma cells exhibit both epithelial and mesenchymal phenotypic features concurrently (390) (Fig. 6). Here, the mesenchymal features enable cell migration from the primary tumor site, and the carcinogenic progeny can transform into epithelial cells at the metastatic site due to the variegated epithelial mesenchymal stage (391). According to Gunasinghe *et al.* (392) and Stankic *et al.* (393), this is essential for the development of micrometastatic outgrowths and the generation of metastatic colonies. Due to the loss of the phenotypic plasticity required to repopulate the tumor at the metastatic site, cells that have completed the EMT mechanism and lost their epithelial characteristics are ineffective at initiating metastatic colonies (394, 395). Cancer cells undergoing MET exhibit a certain level of phenotypic plasticity, making them extremely capable of performing metastasis (393, 396). Partial EMT is a crucial mechanism because cells do not have to transition to a mesenchymal state to benefit from the EMT pathway.

### EMT and MET in CSC formation

There is a strong correlation between cancer progression, stemness, and EMT. Cells that acquire the stem-like phenotype after EMT typically become more aggressive (397). According to Garg *et al.* (398), CSCs can have two distinct phenotypes: mesenchymal with increased migratory and invasive potential, or epithelial with enhanced self-renewal and differentiation potential. Together with the TME, EMT helps CSCs locate the secondary tumor. EMT also plays a role in transforming non-CSCs into CSCs (398). Following EMT induction through the RAS/mitogen-activated protein kinase pathway, non-stem cell epithelial cells in breast cancer were found to exhibit stem-cell characteristics (399). TGF-β–induced EMT causes cells to express NANOG and SNAIL1 more frequently, resulting in stem-like characteristics (400).

Stem cells isolated from different cancers have been found to express EMT-specific markers, while infiltrated cancer cells have been found to express CSC markers (401, 402). This may indicate that EMT and CSCs are closely related. According to several studies, MET and stemness in cancers are linked. To colonize and micrometastasize, dispersed cells must transform from mesenchymal to epithelial cells while retaining their stemness (401). However, it is unclear how epithelial cells recover their stemness after MET. Research has shown that downregulating EMT-transcription factors, like *PRRX1*, can cause stemness (361); however, traditional transcription factors, like *TWIST,* do not exhibit the same effect (403, 404).

The relationship between CSCs phenotypes and epithelial status is associated with later stages of metastasis, while the development of the mesenchymal phenotype is associated with early metastasis events (405, 406). CSC creation is an early event in cancer progression. Furthermore, it appears that epithelial-mesenchymal heterogeneity and plasticity remain closely related throughout the metastatic process, as advanced cancers exhibit a significant increase in both plasticity and the expression of transcription factors that regulate the interconversion between epithelial and mesenchymal states (407, 407).

Additionally, a recent study by Sylvia K. Plevritis *et al.* (408) that clarified the range of MET and EMT states in clinical samples offers insights into the course of cancer and drug resistance. Through the use of mass cytometry time-course analysis, they are able to resolve lung cancer EMT states by treating the cancer with TGFβ and distinguish a unique MET state by withholding TGFβ. Utilizing a computer program to recreate the paths between different cell states (TRACER) (408), they show significant differences between EMT and MET trajectories. Moreover, we create the EMTMET PHENOtypic STAte MaP (PHENOSTAMP), a reference map of EMT and MET states for lung cancer (408).

For *in vitro* EMT-MET analysis, they project clinical samples onto the EMT-MET PHENOSTAMP using a neural net algorithm to provide single-cell resolution phenotypic profile characterization. Together with the *in vitro* EMT-MET results, they offer a framework for phenotypically characterizing clinical samples that may be used in future research to evaluate the clinical applicability of EMT in cancer (408).

To summarize, this framework provides a method for phenotypically characterizing clinical samples in relation to the results of EMT-MET *in vitro*, which may aid in determining the clinical significance of EMT in cancer research in the future.

### Metabolic plasticity

The metabolic reprogramming observed during cancer development is explained by concepts of metabolic flexibility and plasticity as adaptations to energy demands (409). The ability of cancer cells to use various nutrients, such as glucose, lactate, asparagine, and alanine, to support cancer growth and survival is known as metabolic flexibility (410). The term “metabolic plasticity” describes the capacity of malignant cells to respond to environmental stimuli and stresses by dynamically switching between glycolysis and oxidative phosphorylation (410, 411).

Although aerobic glycolysis serves as the main energy source for cancer cells, metabolic flexibility plays a crucial role in conferring survival benefits to cancer cells that encourage metastasis, carcinogenesis, and therapy resistance (412, 413, 414). Glycolysis and oxidative phosphorylation (OXPHOS) phenotypes in cancer cells result in the production of biomass and energy. Cancer cells with a combined phenotype have better survival rates against foreign stimulants and are metabolically flexible (415, 416).

A mathematical model that integrates hypoxia-inducible factor-1 (HIF-1), ROS (both cytosolic and mitochondrial), and 5′-AMP-activated protein kinase (AMPK, a key regulator of mitochondrial respiration and biogenesis) was created using a systems biology modeling approach to comprehend the transition between phenotypes (417). ROS play a major role in the AMPK–HIF-1 switching mechanism (418). Computational modeling of these circuits demonstrates that cancer cells can differentiate into any of the three safe phenotypic states (1): glycolytic, characterized by low AMPK activity and high HIF-1 (2); OXPHOS, characterized by low AMPK activity and high HIF-1; and (3) mixed glycolysis/OXPHOS, which arises when both HIF1 and AMPK levels are elevated (418). This framework shows that high rates of ROS production in the mitochondria, HIF-1 stabilization, and c-SRC, RAS, and MYC regulation are responsible for the hybrid phenotype. It is thought that this hybrid phenotype plays a significant role in cancer maintenance as it has demonstrated high proliferative and clonogenic abilities in tumor models (418, 419, 420). CSCs benefit from multiple adaptive advantages due to their metabolic plasticity. The expression of metabolic glycolytic genes, such as c-MYC, GLUT-1, and PDK1, has been shown to promote CSC proliferation in the presence of glucose (421). However, they activate PGC1-α, a peroxisome proliferator–activated receptor gamma coactivator, which enables the switch to OXPHOS for energy generation when glucose levels become low (422, 423). It has been proposed that this metabolic shift plays a significant role in CSC survival after metastasis. CSCs exhibit metabolic plasticity by adjusting to different energy sources, such as fatty acids, glutamine, ketone bodies, lactate, and methionine, under different stress conditions (424). Moreover, metabolic plasticity encourages EMT. A higher rate of glycolysis is seen in cancer cells going through EMT, and this is associated with anabolic metabolic pathway suppression. A range of metabolites associated with EMT may influence cellular plasticity by altering lipid and amino acid metabolism and epigenetic regulation (285, 425).

Particularly, it has been proposed that the hypoxic microenvironment is crucial for preserving lung CSCs, which raises the malignancy of lung cancer (426). HIF-1α and HIF-2α, which both have the ability to control lung CSCs stemness and encourage chemoresistance, are the primary factors associated with the hypoxic microenvironment (427). Furthermore, transcription factors SOX2 and Oct4 support the preservation of lung CSCs stemness, particularly in hypoxic environments. Using *SOX2* and *OCT4*, for instance, which have been shown to be transcription factors significantly involved in the formation of induced pluripotent stem cells, Iida *et al.* (2012) (428) discovered that both these proteins regulated lung cancer malignancy (429). Furthermore, Hua *et al.* 2020 (427) reported that HIF1α and HIF2α can also control the stemness of CSCs and make them more chemoresistant. Nonetheless, the majority of research has concentrated on controlling hypoxia in CSCs; little is known about how hypoxia affects differentiated lung cancer cells. Conventional research has demonstrated that, in hypoxic environments, differentiated lung cancer cells can undergo dedifferentiation to produce lung CSCs (430); however, the precise mechanism underlying this process remains unclear. Yan Li's 2023 study (431) reveals that in a hypoxic microenvironment, HIF1α and HIF2α control NSCLC chemoresistance by dedifferentiating through *SOX2* and *OCT4* expression. This discovery unveils a novel pathway for lung cancer and offers fresh avenues for treating the disease and its progression. It was demonstrated that under hypoxic conditions, the formation of spheres by differentiated lung cancer cells was higher than that by control cells under physiologic conditions.

CD133 and CD44 were strongly expressed in these freshly formed spheres. The Cancer Genome Atlas database revealed that lung cancer tissues had elevated expression levels of HIF1α and HIF2α. The expressions of SOX2, OCT4, CD133, and CD44 significantly decreased after knocking out HIF1α and HIF2α, and the expressions of CD133 and CD44 decreased after knocking out SOX2 or OCT4. In conclusion, under hypoxic conditions, SOX2 and OCT4 regulate the dedifferentiation of NSCLC by HIF1α and HIF2α.

The TME and transcription factors are common to both CSCs and normal stem cells and impact the transition between CSCs and non-CSCs (401, 432) (Fig. 5). The TME encourages metastasis and relapse while supporting CSC self-renewal, differentiation, and maintenance. Recent evidence has indicated a possible role of the TME in regulating and inducing plasticity through different cellular interactions and signaling molecules. Numerous cell types found in the niche, such as immune cells, exosomes, MSCs, and CAFs, significantly contribute to CSC plasticity (345, 433).

Two cytokines produced by macrophages, oncostatin-M and osteopontin, enhance the capacity of CSCs to form colonies and to dedifferentiate non-CSCs into CSCs, respectively (434, 435). Through the *Notch, IGF-II/IGF1R, c-Met/FRA1/HEY1*, and *FAK* signaling pathways, MSC-derived fibroblasts and CAFs influence plasticity in a variety of malignancies (436, 437). Exosomes regulate the conversion of non-CSCs to CSCs, assist in stem cell regeneration, and encourage CSC self-renewal *via* the Notch, Wnt, and Hh signaling pathways. Exosomes can be secreted by stromal cells or be derived from non-tumor and tumor cells or from CSCs (438, 439). Hypoxia and acidosis linked to niches promote plasticity by controlling stemness genes, such as *OCT-4, SOX-2,* and *NANOG* (285, 433, 434).

## Factors leading the TCP

Targeted drug resistance remains a major obstacle to complete recovery even in cases of cancer, despite the success of these treatments (435, 436). By changing their phenotype, tumor cells can recur despite treatment, due to intrinsic or exogenous cell plasticity (437). TCP can be circumvented by several reversible mechanisms, such as epigenetic modifications, transcription factor regulation, the activation or suppression of important signaling pathways, and TME modification (437). Tumor cell and CSC formation, as well as the EMT, lead to TCP (438).

Corresponding and combined therapy techniques have been developed that concentrate on circuits associated with plasticity (439). It has also been addressed how targeted drugs cause TCP in a variety of tumor types through nongenetic mechanisms, providing insight into the role that TCP plays in acquired drug resistance (439, 440). Novel approaches to treatment have been discussed, including ways to prevent or reverse the ability of tumor cells to adapt (441).

An important obstacle to attaining the intended therapeutic outcomes is therapy-induced drug resistance, even though numerous drugs have been developed to help patients with cancer live longer and have better prognoses. Up to 90% of cancer-related fatalities are caused in part by drug resistance (442). Acquired drug resistance is a consequence of targeted treatments and is linked to TCP and the tendency of cancer cells to play “hide-and-seek” (438). The drug-resistant phenotype of cancer cells is temporary and reversible rather than an inherited trait (438). Having a thorough understanding of the mechanisms behind induced plasticity should aid in the creation of innovative treatments (443). The TME, essential signaling pathways, transcription factors, and epigenetic changes may all play a role in the therapy-induced plasticity of various malignancies (443).

A significant factor in TCP’s resistance to targeted therapy is a network of transcription factors (438). During the EMT process, EMT transcription factors, such as *TWIST* and *SNAIL*, actively respond to targeted therapy, which leads to improved mesenchymal switching and plasticity (444). In addition to these EMT-related transcription factors, TCP is regulated by several other transcription factors (445). Androgen receptors (ARs), which are also known as ligand-activated transcription factors, regulate which genes are expressed in the prostate epithelium (445, 446).

For prostate cancer treatment, AR-targeted inhibitors, such as enzalutamide and bicalutamide, have been developed (447). However, following castration therapy, point mutations, splicing variants, AR amplification, and the replacement of AR functions by glucocorticoid receptors all set off evading mechanisms (447). An extensively studied transcription factor in various cancer types is the *STAT3* (448). Trastuzumab and trastuzumab-emtansine resistance may be connected to human EGFR 2 expression in breast tumors that activate *STAT3* (HER2) (448). After receiving trametinib therapy for 7 days, 117,118 patients with ER−/PR−/HER2− TNBC demonstrated a significant transcriptomic response, with up to 22% of the transcriptome displaying notable upregulation or downregulation (449). Myc was quickly degraded during the treatment but persisted in influencing the recruitment of transcriptional complexes and genomic reorganization, which, in turn, contributed to tumor phenotype changes (443, 450).

Numerous signaling pathways contribute to embryonic development. Tumor growth and treatment resistance depend on the reactivation of these pathways (451). One important way that conserved signaling pathways, such as the Hh, Wnt, and NOTCH pathways, interact with other oncogenic pathways, such as the NF-κB, PI3K/AKT/mTOR, and MAPK pathways, is through the regulation of downstream effectors (Fig. 1) (452). One major obstacle to the efficacy of tumor therapy protocols is phenotypic switching caused by different signaling pathways. One-third of cancers have abnormalities in the Hh signaling pathway, and the dysregulation of this system has a major effect on treatment resistance (452, 453). Myeloma cells resistant to belumumab demonstrate abnormal Hh signaling activation, followed by an increase in the downstream effector Gli2. Scientific research has demonstrated that suppressing Hh signaling can cause CSCs differentiation and that Hh signaling is necessary for the self-renewal of CSCs in multiple myeloma (454). In TNBC, CAFs can be reprogrammed to support a shift in CSC phenotypes and the acquisition of chemo-resistant characteristics *via* the Hh ligand generated by tumor cells (455). TCP can be reduced by reducing CSCs proliferation, metastasis, and the EMT program, which can be achieved by successfully blocking the Hh signaling pathway (456).

The Wnt signaling pathway and TCP have a strong relationship. Wnt signaling is a complex mechanism that may function in two ways, autocrine or paracrine, through noncanonical routes, such as the planar cell polarity pathway, or canonical pathways such as the Wnt/β-catenin pathway. Wnt signaling helps regulate TCP and EMT (438). The Wnt/β-catenin signaling pathway usually causes the EMT regulator SNAIL to be expressed more when it is constitutively active. It has been demonstrated that in a colorectal cancer model, EMT and tumor metastasis can be avoided by blocking the Wnt/β-catenin pathway (438). Wnt/β-catenin–driven downstream protein invasion of a tumor may be mediated by *SLUG*, another EMT modulator. According to Moon *et al.*, concurrent expression of both Slug and β-catenin is linked to increased survival and lymph node metastasis in individuals with head and neck squamous cell carcinoma (457).

Slug overexpression can increase cell invasion and metastasis, as well as induce cancer cell stemness. According to Sun *et al.*, Slug overexpression is linked to liver cancer invasiveness in both *in vitro* and *in vivo* settings (458). However, liver cancer cells do not show similar invasiveness in response to *SNAIL* overexpression. *TWIST* is another EMT transcription factor that has demonstrated increased expression in mammary epithelial cells upon Wnt pathway activation (459). A growing body of evidence indicates that TWIST promotes CSC growth, accelerates the rate of carcinogenesis, and induces EMT (459).

The most effective way to prevent cancer spread and recurrence may be to inhibit these EMT mediators. The Wnt signaling pathway regulates the stemness of CSCs in several cancer types, such as HCC, lung, colon, and other malignancies (451, 460). Clinical experience shows that heightened tumor recurrence and unintentionally elevated Wnt activity are the outcomes of targeted therapy with MEKi (438). Similarly, in breast cancer, the HDAC inhibitor valproic acid can increase the population of CSCs and stimulate the Wnt signaling pathway, which can accelerate tumor growth (461). Therefore, unexpected phenotypic flipping mediated by the Wnt signaling system should be considered during targeted treatment.

Malignant mesothelioma, lung cancer, pancreatic ductal AC, and breast cancer are among the cancers for which the Notch signaling pathway stimulates CSCs and controls the EMT program (162, 438). A previous study discovered that the EMT status of lung ACs was changed by gefitinib therapy (462). Further research provided evidence that gefitinib-acquired resistance develops because of Notch1 signaling pathway activation during the EMT process (463). In NSCLC, one of the key players in neuroendocrine transdifferentiation regulation is Notch signaling, which is one of the main regulators of neuroendocrine transdifferentiation. This pathway makes it possible for NSCLC to advance to SCLC and acquire EGFR-TKI resistance following initial targeted therapy (464).

The Notch pathway is crucial for the EMT process in prostate cancer cells, promotes the transition to basal stem-like traits, and produces a phenotype that is resistant to castration (465). Tumor-invasive regions exhibit significant expression of Notch signaling pathway components, indicating the pathway’s critical role in the regulation of EMT. According to Natsuizaka *et al.*, Notch1 signaling and EMT work together to encourage the development of tumors that lead to squamous cell carcinoma. Notch1 is directed to drive EMT by TGF-β, another significant protein (466, 467). According to Saad *et al.*, the control of EMT transcription factors is linked to Notch-mediated EMT activation (468, 469). *SNAIL* and *SLUG* are two transcription factors that contribute to the Notch pathway's inhibition of β-catenin and E-cadherin (469).

The inhibition of Notch activation reduces the expression of SNAIL and restricts the expression of MMP-9 and MMP-2. Research has unequivocally demonstrated that Notch signaling causes EMT activation *via* the SNAIL pathway (467, 468). The possibility that Notch signaling could accelerate the EMT process by collaborating with other pathways is unexpected (467, 468). The suppressor of mothers against decapentaplegic (SMAD) proteins regulates the expression of the genes necessary for the transition to mesenchymal features through their interactions with other transcription factors. This interaction facilitates crosstalk between these pathways. The single-cell sequencing work by Deshmukh *et al.* revealed that the activation of EMT signaling pathways occurs both sequentially and simultaneously, and the Notch pathway is essential for regulating TGFβ-induced EMT (469).

### TME-driven TCP regulation and targeted therapy resistance

TCP is significantly impacted by TME brought on by treatment. Key TCP regulators include cytokines, exosomes, immune and stromal cells, hypoxia, pH variations, malnourishment, and angiogenesis. The communication that takes place between the stromal cells that encircle the tumor cells is known to be greatly aided by CAFs (470).

Through the production of diverse bioactive molecules, including cytokines and growth factors, CAFs significantly impact TME and TCP and also play a role in EMT (470, 471). CAFs also influence the growth of the CSC pool (472). Apicella *et al.* (473) has been proven that tumor adaptive resistance to a MET TKI (erlotinib) in an vivo study on mice is driven by stroma, and in particular by CAFs This type of resistance is a non-autonomous resistance, because it requires a constant stimulation of the compound otherwise even the cell derived from the xenograft mouse model were no more resistant to TKI. vitr. CAFs could explain this kind of resistance. The body’s metabolism is altered by erlotinib administration *in vivo*, favoring aerobic glycolysis and increasing lactate production. When this lactate is released, hepatocyte growth factor (HGF) is overproduced by CAFs. Erlotinib resistance in cancer cells is caused by the MET signaling pathway, which is activated by consumed lactate and the subsequent increase in HGF (473). Furthermore, hypoxia-induced glycolysis forms an immunosuppressive TME by reducing cytotoxicity through decreasing NKG2D, CD16, perforin, and granzyme B levels and suppressing natural killer cells through lactate accumulation (474).

It has been proposed that therapeutic monoclonal antibody resistance is influenced by these hypoxia-related TME situations (438). Additionally, cytokines released by immune and stromal cells offer hints regarding acquired TCP brought on by targeted treatment (438). Li *et al.* report that osimertinib therapy resulted in a significant increase in IL-6 levels in patients with EFGR-mutant NSCLC (475). Additionally, there was an associated upregulation of LAMININ α5, one of the LAMININS that is most widely distributed and plays a significant role in the ECM. Acquired resistance against Osimertinib was facilitated by IL-6’s remodeling of ECM and activation of a downstream target focal adhesion kinase (FAK) through upregulation of laminin α5. It has been established that FAK activation is not necessary for the production of IL-6 during the upkeep of mesenchymal stem cells and may also result in acquired gefitinib resistance (438, 475).

Targeted therapy is one of the best therapies available for addressing the fundamental causes of several malignancies (476). However, the development of drug resistance raises the risk of cancer and severely reduces the effectiveness of targeted medications (476). Drug target mutations and bypass pathway activation provide cancer cells with resistance to targeted inhibitors (477).

#### TKI resistance: a persistent issue

I.B. Weinstein (478) first described the phenomenon known as "oncogene addiction" in 2002 (479). He describes how cancer cells grow unduly reliant on a specific "driver" mutation in order to survive. These types of dependencies make cancers extremely vulnerable to medications known as "targeted therapies," which block the drivers. Targeting particular molecular subsets of cancer has proven to be highly successful over the last 10 years in a variety of human cancers, most notably NSCLC. The first driver changes identified in NSCLC were somatic activating mutations in the EGFR (EGFR), which affect 10 to 15% of non-Asian patients (480). These mutations cause high response rates and prolonged progression by making EGFR sensitive to small molecule TKIs (481, 482, 483, 484, 485, 486). An equivalent level of clinical benefit from anaplastic lymphoma kinase (ALK)-TKIs is predicted by rearrangements involving the *ALK* gene, which are found in 3 to 7% of NSCLC cases (487). With advanced NSCLC, genotype-directed therapy is currently the standard of care and has improved overall survival (488, 489).

Today's ubiquitous challenge for long-term disease control is resistance, which inevitably limits the efficacy of TKI therapy and dampens enthusiasm. At its foundation, cancer is a microcosm of evolution. Selective pressures from TKI therapy influence the long-term accumulation of mutations and genetic diversity, which are the main factors driving its survival. TKI resistance, which is typically classified as primary (intrinsic) or secondary (acquired), is refractory due to these simple yet complex principles. When a patient exhibits primary resistance, they do not respond to targeted therapy. Patients who develop secondary resistance first experience some clinical improvement before their illness worsens. A greater variety and quantity of resistance mechanisms are being identified with the identification of every oncogenic driver and targeted inhibitor.

Reviewing randomized studies using EGFR- or ALK-TKIs in the first-line context for advanced EGFR- or ALK-positive NSCLC, respectively, revealed that 4 to 10% of newly diagnosed patients experience primary resistance, which is defined as "progressive disease as the best response" (481, 482, 483, 484, 485, 486, 487).

Although the exact mechanisms behind intrinsic TKI resistance are unknown, they may involve nonsensitizing changes in the target. For instance, 4 to 10% of EGFR mutations are caused by in-frame exon 20 insertions (488, 489, 490). *In vitro*, these stimulate EGFR signaling, but they do not make first-generation EGFR-TKIs–sensitive (488, 489, 490, 491). A widely recognized mechanism of acquired resistance to initial-generation EGFR-TKIs is the gatekeeper mutation, or EGFR T790M. Patients without prior treatment who have classic activating EGFR mutations may develop intrinsic resistance if preexisting EGFR T790M-mutant clones are present, provided that the allelic frequency threshold is met (492, 493). Preexisting T790M mutation frequencies have been reported in the literature at varying frequencies (less than 10% to 65%), depending on the approach of detection (492, 493, 494).

Reduced *de novo* sensitivity to TKIs can also be caused by genetic changes that occur outside of the target kinase. Prior to TKI exposure, MET amplification has been documented in EGFR-mutant NSCLC (495). *BIM*, also known as *BCL2L11*, is a BCL2 family member that mediates apoptosis that is brought on by a number of TKIs. According to recent research, EGFR-TKI efficacy is correlated with lower levels of *BIM* mRNA expression (494, 495, 496, 497, 498). Furthermore, a BIM polymorphism that produces isoforms devoid of the proapoptotic BH3 domain was linked to a suboptimal response to EGFR-TKIs in Asian patients (497).

For other oncogene-driven lung cancers, there is presently no evidence that BIM is as important. EGFR-TKI–induced intrinsic NSCLC sensitivity may also be modulated by NF-κB. By RNAi-mediated knockdown of NF-κB pathway components, EGFR-mutant lung cancer cells became more sensitive to erlotinib; similar outcomes were obtained with pharmacologic inhibition of NF-Κb (498). In 52 patients with EGFR-mutant lung cancer receiving erlotinib treatment, higher expression of the NF-κB inhibitor IκB was associated with a better response to therapy and a higher survival rate (498). This implies that blocking both the NF-κB pathway and EGFR may improve the way that EGFR-TKIs work in clinical settings.

Primary TKI resistance–inducing gene alterations have been connected to the induction of EMT (499, 500). To illustrate, Park *et al.* showed that *SRC* activation mediated erlotinib resistance conferred by overexpressing *CRIPTO-*1, an EGF-CFC family member, in cell line and mouse xenograft models (500). In 85 NSCLC patients with EGFR-sensitizing mutations, higher levels of Cripto-1 expression were associated with intrinsic resistance to EGFR-TKIs (500). To validate these findings and ascertain whether Cripto-1 is a noteworthy modulator of EGFR-TKI sensitivity in clinical contexts, more investigation is required.

Finally, at least some cases of apparent primary resistance may be explained by false-positive genotyping. The gold standard for *ALK* rearrangement diagnosis has been the break-apart FISH assay. A minor intrachromosomal inversion event causes the most prevalent *ALK* rearrangement, EML4-ALK, which occasionally causes a slight separation of the 5′ and 3′ signals (501). Significant interobserver variability and possible false-positive results may result from this (501). Thus, next-generation sequencing or immunohistochemistry can be useful for secondary confirmation of ALK positivity.

The growing body of knowledge about TCP indicates that it is a major mediator of drug resistance (438). The main topic of this section is how TCP influences tumor cell characteristics and mediates resistance to targeted treatments (440). As mentioned earlier, EMT acts as a mediator between TCP and resistance to targeted therapy (440).

The three main mechanisms of EMT-related drug resistance are escape from apoptosis, increased drug efflux, and steady cell growth (477). The most researched cancer subtype that links targeted medication resistance and EMT is lung cancer (477). The overexpression of the EMT-related gene AXL has been shown by Namba *et al.* to promote the development of osimertinib-resistant acquired resistance. Additionally, TKI resistance has been connected to other EMT-related transcription factors that regulate EMT-related signaling pathways, including *SNAIL*, *SLUG*, and *ZEB1/2* (438, 477).

To avoid targeted therapy resistance, it is essential to fully comprehend the EMT process. Signaling pathways such as Notch, Wnt/β-catenin, and TGF-β are crucial for triggering transcription factors associated with EMT (502). Based on available information, EMT transcription factors may contribute to resistance to targeted treatments, especially when it comes to EGFR inhibitors. Consequently, drug resistance is mediated by the same signaling pathways that control the EMT process. Chang *et al.* found that SLUG suppresses CASPASE-9 activity and downregulates BIM, both of which increase the resistance of NSCLC cells to gefitinib (503).

#### TGF-β: an important EMT inducer

TGF-β is an important EMT inducer due to its ability to strongly induce EMT and its involvement in cancer-associated EMT (504). Cellular processes, such as glycolysis and lipid/choline metabolism reprogramming, depend on TGFβ-induced EMT (505). Cancer cells can exhibit either a full or partial EMT in response to TGF-β, exhibiting concurrent mesenchymal and epithelial markers. The incomplete EMT condition is characterized by an aggressive phenotype that confers strong stemness and cell plasticity to cancer cells. Following TGF-β receptor activation, TGF-β signaling pathways are classified as SMAD-dependent or SMAD-independent (506). TGF-β/SMAD and non-SMAD pathways play a key role in promoting cell plasticity and mediating EMT (506, 507). It has been documented that TGF-β–induced EMT depends on signaling pathways, such as mTOR, MAPK, and PI3K/AKT (506, 507). Remarkably, EMT caused by TGF-β may be reversed. According to a study by Katsuno *et al.*, mesenchymal cells revert to the phenotype of epithelial cells when TGF-β is removed (506, 507, 508).

According to preclinical research, TGF-β has been demonstrated to be important at the pulmonary level in pathological conditions like lung cancer as well as physiological ones like lung organogenesis (509, 510). In particular, this cytokine is the main inducer of the reversible physiological process known as EMT, which is implicated in the growth, dissemination, and metastasis of tumors (511). An increasing body of research indicates that TGF-β and EMT play a critical role in the progression of LC (512, 513, 514). As such, a deeper comprehension of the molecular mechanisms underlying these processes may yield important predictive markers and therapeutic targets for LC.

Long-term exposure to TGF-β induces cancer cells to undergo a permanent EMT process, which has been linked to a rise in tumor stemness and resistance to treatment. Researchers have proposed that because mTOR inhibitors impede the TGF-β–induced EMT process, they might be able to target CSCs.

#### EMT-related miRNAs affect drug resistance and cancer cell stemness

Small noncoding RNAs known as miRNAs are essential for controlling the processes that lead to tumor metastasis through EMT and metastatic colonization through MET (508).

EMT-related miRNAs affect drug resistance and cancer cell stemness. According to recent research, miRNAs may influence the EMT process by focusing on associated transcription factors, such as *SNAIL*, *SLUG*, *TWIST*, and *ZEB1/2* (515). Several metastasis-related miRNAs, such as miR-203, miR-204, miR-34c, and miR-153, may regulate the expression of SNAIL (516). In several cancer subtypes, including breast and lung cancer, the miR-30 family of molecules is a well-established modulator of *SNAIL* (257). Liu *et al.* demonstrated that mesenchymal differentiation and EMT are inhibited by miR-1 and miR-200 *via* Slug-dependent pathways (477).

While some recently identified miRNAs, such as miRNA-490-3p, directly encourage the spread of invasive ductal carcinoma, others, including miR-27b3p, have been linked to EMT *via* the stimulation of circulating tumor cell generation (517). However, several miRNAs, such as miRNA-128 and miRNA-155-5p, can obstruct a number of signaling pathways associated with EMT, consequently impeding it (517). Given the role of miRNAs in EMT and TCP, cancer drug resistance could be overcome by employing various strategies aimed at impeding miRNA function or miRNA delivery into tumor cells (518).

As a result, EMT is associated with the regulation of miRNA expression; in NSCLC cell lines and tissues, it was discovered that miRNA-330-3p and miRNA-205 were upregulated and downregulated, respectively, and it has been shown that TGF-β–induced EMT in NSCLC cells can be controlled by miRNA-330-3p inhibitors or miRNA-205 mimics (519). Furthermore, in NSCLC, miR-330-3p promoted cell invasion and metastasis, through the induction of EMT, while miR-205 inhibited EMT to limit NSCLC (519). miRNA-16 has also been linked to pulmonary tumorigenesis, specifically in lung AC, where it has been demonstrated that overexpression of transcription factor *AP-2α* causes EMT through the miR-NA-16 family/transcription factor *AP-2α* /PSG9/TGF-β (520).

It has also been demonstrated that QKI-5, a significant protein involved in RNA signal transduction, decreased in metastatic lung AC. Furthermore, overexpression of this protein *via* TGFβRI prevents TGFβ-induced EMT-mediated invasion and metastasis (521). Furthermore, it has been noted that the TGF-β pathway is influenced by the interaction between LIM and SH3 domain protein 1, two recently discovered metastasis biomarkers, *via* phospho-SMAD2/3 and SNAIL1 localization regulation. Vimentin, N-, and E-cadherin are examples of EMT markers whose expression is impacted by this (522).

A fascinating analysis of the immune landscape in NSCLC using EMT scores showed that EMT was associated with increased levels of immunosuppressive cytokines such as TGF-β and a markedly decreased infiltration of CD4 T-cells in lung AC and CD4/CD8 T-cells in squamous cell carcinoma (523). Further analysis showed that in alveolar type II epithelial cells treated with 4-OHT or TGF, respectively, TGF-β and RAS activation induce total and partial epithelial migration (524).

TGF-β has been shown to EMT and the acquisition of cancer stemness in human lung cancer (A549) and normal lung epithelial (BEAS-2B) cell lines (525). This is accomplished by demethylating of the promoters of Slug and CD87, activating them. Furthermore, it was recently observed that EMT induction in lung cancer has been connected to the development of lung cancer and could be a major effect of nickel exposure (526).

In NSCLC A549 cells, TGF-β or ginsenosides Rk1 and Rg5 treatment inhibits the Smad and NF-kB/ERK pathways (non-Smad pathway) to control EMT in a dose-dependent way (527). In A549 cells, ginsenoside CK prevents TGF-β–induced EMT and metastasis (527); additionally, ent-caprolactin C, a novel compound, suppresses the EMT cell marker proteins and phosphorylates Smad2/3 in TGF-β–treated A549 cells, thereby inhibiting TGF-β–induced EMT and potentially acting as an antimetastatic agent (528). Furthermore, prolyl 4-hydroxylase α3, an enzyme linked to cancer metabolism, is necessary for the TGF-β–induced reprogramming of amino acid metabolism. Tumor metastasis results from TGF-β–dependent alterations in amino acid inhibition and EMT, which are promoted by downregulating prolyl 4-hydroxylase α3 (529).

Finally, in NSCLC cell lines (A549 and SPC-A1), TGF-β positively regulated N-cadherin, triggering the SMAD3/4 complex (530). Furthermore, through the JAK2/STAT3 and SHP2/Grb2/PI3K/AKT signaling pathways, cancer cell anoikis resistance, invasion, and EMT were promoted by decreased SH2B3 and elevated TGF-β in LC (531). Another interesting protein that is overexpressed in NSCLC and has been linked to bone metastases is the formin-like 1 protein. Cells' ability to migrate, invade, and spread is reduced when TGF-β/SMAD-mediated EMT is inhibited in formin-like 1-knockdown A549 and PC9 cells as well as in mice (532). It's also noteworthy that TGF-β type III receptors and the high mobility group protein A2 compete for the let-7 miRNA family, which results in EMT and pulmonary cancer metastasis (533).

Additionally, it has been shown that EMT inhibition makes LC patients more susceptible to EGFR-targeted therapy (534). When considered collectively, the increasing amount of data indicates that TGF-β–mediated EMT is a well-studied process linked to lung cancer and identifies a number of potential contributing factors to the development and progression of lung cancer.

### Tumor heterogeneity

The existence of a subset of cancer cells inside the primary tumor that possess distinct phenotypes, genotypes, and a range of other characteristics is referred to as tumor heterogeneity. Different susceptibility to therapy and the emergence of medication resistance may result from heterogeneity (535, 536). The CSCs plasticity model is the most recent model of tumor heterogeneity (357).

CSCs have the ability to self-renew and transition between stem and differentiated forms. Changes in TME, epigenetic modifications, and gene mutations are responsible for this dynamic transition. The CSC transition promotes drug resistance, metastasis, and cancer recurrence and is mediated significantly by EMT signaling pathways (537). According to Morel *et al.*, inducing EMT can increase the stemness and tumorigenic properties of human breast epithelial cells, a finding that has been verified in colorectal, pancreatic, and liver cancers (538).

Quintana *et al.* demonstrated that the phenotypic plasticity hypothesis may account for most of the phenotypic variation observed in melanoma (539). Despite the lack of a reliable marker to distinguish tumorigenic from nontumorigenic cells, CD271+ cells showed low tumorigenic activity. Other studies have demonstrated that the contribution of cell plasticity to tumor heterogeneity can differ based on the type of cancer (540). Charles *et al.* found that nitric oxide may increase the ability of glioma cells stimulated *in vivo* by platelet-derived growth factor to proliferate tumors in a reversible manner (541). In the development of colorectal cancer, Nakano *et al.* described the dynamic equilibrium between CSCs and nonCSCs (542, 543). This equilibrium is primarily regulated by the EMT-associated TGF-β signaling pathway (272). According to one study, colon cancer cells can dedifferentiate into CSCs when they acquired the LGR5+ phenotype (544). They found that most of the circulating colon cancer cells were LGR5− and in contrast the cells in the metastases were LGR5+ CSCs.This finding emphasizes the unique role that CSCs play in the growth of primary and metastatic tumors (544). Numerous studies have revealed that poor responses to targeted medications, such as gefitinib, rociletinib, and osimertinib, are linked to a high degree of tumor heterogeneity (438, 545, 546).

According to Chabon *et al.*, 46% of patients experience multidrug resistance following targeted therapies, indicating a high frequency of intratumor heterogeneity (546) Selective pressure associated with therapy, TCP, or phenotypic switching between various signaling pathways may result in heterogeneity. Several critical biological processes, such as tissue regeneration, wound healing, and embryonic development, depend on cell plasticity. The reactivation of these mechanisms in tumors results in drug resistance, metastases, and tumor progression as tumor cells switch between non-CSC and CSC-like phenotypes (547).

Using a whole genome analysis, Easwaran *et al.* found that blocking cell clones with abnormal self-renewal capacity and suppressing aberrant promoter DNA methylation can reduce population plasticity changes (548, 549). Thus, disrupting TCP could be a viable method to address tumor heterogeneity and circumvent drug resistance (436, 437). Cancer cells and their surroundings have intricate relationships with TME, which is composed of blood vessels, tumor, immunological, stromal, and ECM cells (450). A growing body of research indicates that cancer cells use intricate signaling pathways to regulate the operation of cellular and noncellular components (485). Nonmalignant cells are pushed to take on new characteristics by interactions with the ECM that encourage tumor cell growth, division, and metastasis. CE, tumor heterogeneity, and drug resistance are all mediated by the interactions between cancer cells and the TME (438, 550, 551).

TCP, which is mediated by the TME, allows tumor cells to alter their phenotypes and resist therapy using adaptive methods (552). Oxidative stress is a key mediator of several adaptive strategies and is influenced by both hypoxia and CAFs. Hypoxia is a defining feature of the TME and is highly associated with poor clinical responses, therapy resistance, and malignancy progression (553). Hypoxia has been linked to phenotypic switching and subsequent drug resistance (553). Terry *et al.* reported that hypoxic stress alters EMT transcription factors, such as *ZEB2*, *SNAIL1*, and *SNAIL2*, and leads to phenotypic diversity in lung cancer. These changes result in cell-mediated cytotoxicity resistance (554, 555). Paolicchi *et al.* stated that tumor hypoxia may play a significant role in treatment resistance and be associated with an increased risk of CSC development and metastasis (556). Thus, blocking proteins linked to EMT, mitochondrial functions, and HIF-1α could be one way to treat cancer since it would prevent the body's reaction to hypoxia. Furthermore, it's thought that cytokines like TNFα and interferon-gamma improve stromal cell pliability and activate the TME (557).

Genetic heterogeneity may be significantly influenced by the relationship between TME and TCP (558). Reynolds *et al.* reported that significantly more genetic mutations occur in tumor-containing cells than in laboratory cultures (559). Their findings offer compelling proof that the TME encourages genetic instability as tumors progress. Tumor heterogeneity may result in TME reprogramming and cancer drug resistance (560). The interaction between TCP and TME plays a critical role in mediating drug resistance, tumor heterogeneity, and tumor progression (560, 561).

Searching for tumors with multiple types of differentiation provides further evidence of the histological and cellular heterogeneity found in lung cancer (562, 563, 564). A good illustration would be adenosquamous carcinomas. The cells exhibit heterogeneity at the cellular level, as evidenced by the presence of AC differentiation markers such as TTF1 and CK7, as well as squamous differentiation markers such as CK5/6 or other high-molecular-weight cytokeratins.

This also holds true for pleomorphic carcinomas, which have areas with squamous or AC differentiation along with giant and/or fusiform cells. Additionally, cellular heterogeneity is seen in mixed tumors: small cell lung carcinomas mixed with other lung carcinomas, like squamous cell carcinomas or ACs, or large cell neuroendocrine carcinomas mixed with other carcinomas. Different cell origins could account for some of this cellular heterogeneity. Certain types of lung carcinomas have defined preneoplastic lesions (564).

One repair/carcinogenic pool of adult stem cells associated with pulmonary carcinoma heterogeneity is the terminal respiratory unit, which comprises the respiratory bronchiole and adjacent alveolar duct/septae (564). The other pool is the adult central respiratory epithelium up to the terminal respiratory unit. These reports have been made. After adult fibroblasts are induced in mouse skin, human-induced pluripotent stem cells' embryonic development shows the entire embryonic potential, providing information on repair and adaptation as well as the potential for meso/ecto/endoderm to mix in a single cell. In this initial investigation, it was shown that CK7, CK5.7, TTF1, VIM, CD56, and Ki-67 were sufficient to categorize carcinomas based on cellular populations and heterogeneity. When fibroblasts and normal bronchial cells come into contact with some environmental toxic agents, like hexavalent chromium, similar outcomes have been seen where the EMT is visible (564).

To summarize, we strongly support the importance of the knowledge of the heterogeneity that may have strong consequences for diagnosis, pathological comprehension, and treatment result against cancer.

### New strategies for targeting TCP

Strategies for targeting TCP include targeting the key nodes of phenotypic switching, combination therapies, and intermittent treatments (438). A study found that vemurafenib-induced melanoma responses could be doubled by sporadic treatments and off-dosing regimens (438). Designing intermittent treatment strategies is typically challenging because drug resistance can be complex and stem from a variety of mechanisms beyond TCP. Combination treatments aim to target the concurrent activation of significant signaling pathways such as EGFR, PI3K, and Hippo/yes-associated protein, (565, 566). Such therapies have demonstrated the ability to significantly decrease the number of remaining tumor cells (551).

Combination treatments are often more complex and require more precisely calibrated drug delivery techniques to minimize side effects and maximize results. Blocking the essential nodes of phenotypic flipping genes has been the focus of TCP prevention strategies (567). The histone demethylases KDM5A/B and KDM6A are important for drug-tolerant persister survival (293, 294, 295). While KDM inhibitors have been created, their specificity and effectiveness are still being evaluated (568).

Drug-tolerant persisters alter their global chromatin landscape as a result of transcriptional adaptation. Metinib resistance has been overcome in TNBC by using a similar strategy that targets the BRD4 gene’s bromodomain and extra terminal domain. This method has proven effective both *in vitro* and *in vivo* (569). Clinical trials treating malignancies that depend on the Wnt and Notch signaling pathways for treatment have not demonstrated success in treating basal cell carcinoma (570). NOTCH-activated tumor cells typically experience apoptosis in response to vismodegib therapy. When combined with MAPK inhibitors, disrupting retinoid X receptor pathways with HX531 can considerably postpone the emergence of MAPK inhibitor resistance compared to using MAPK inhibitors alone. Designing treatment methods will become easier if more pharmacological targets that impede tumor plasticity are identified (438).

The interaction between nontumor and tumor cells during drug exposure results in the formation of an immunosuppressive TME. Immune checkpoint proteins, such as T cell immunoglobulin 3, lymphocyte activation gene 3, cytotoxic T lymphocyte–associated protein 4, programmed cell death 1 receptor (PD-1) and (PD-L1), and T cell immunoreceptor with Ig and ITIM domains (TIGIT), are expressed and play a major role in immunosuppression, acting as a link between EMT programs and immunosuppression (571, 572, 573, 574).

Immune checkpoint proteins and EMT have been found to positively correlate in a number of cancer types, including esophageal, lung, breast, colorectal, and oral cancers. In many different types of cancer, higher expression of these checkpoint molecules is linked to a worse prognosis (573, 574, 575, 576, 577, 578). Since immune checkpoint proteins and therapy evasion are functionally related, immune checkpoint blockade (ICB) in conjunction with other therapies may be a promising way to develop a new tactic against TCP.

Current practice suggests that ICB and targeted therapy can be combined. The application of ICB in EGFR-TKI–resistant NSCLC, particularly in those with upregulated PD-L1 expression, is theoretically made possible by the discovery that traditional EGFR-TKI interferes with TME (579). Further research has demonstrated that TME undergoes dynamic alterations in response to EGFR-TKIs, and it has been proposed that a combination of immune-mediated anticancer strategies and treatment may be advantageous (580, 581, 582, 583). When EGFR-TKI-ICB combination treatment was used instead of pembrolizumab or gefitinib monotherapy, NSCLC patients showed improved clinical benefit, with increased overall response rate (41.7% *versus* 14.3%) and median progression-free survival (19.5 months *versus* 1.4 months) (582).

In fact, combination therapy may be more advantageous for patients with short EGFR-TKI-progression-free survival when used as a second-line treatment (584). Several cancer types were treated with a similar combination approach, known as a "basket trial design." (585). An appealing method of treating HCC is to combine ICB with antiangiogenic molecular targeted therapy. The IMbrave150 trial found that in patients with advanced HCC, atezolizumab plus bevacizumab was more beneficial than sorafenib, and that this combination approach might be utilized as the first-line standard of care (586, 587, 588, 589). Furthermore, ICB and other targeted therapeutic strategies, such as AR blockade and ATR kinase inhibition, worked together to produce a synergistic therapeutic response in prostate cancer (590, 591, 592).

A synergistic therapeutic effect can be extended by combining ICB with chemotherapy (593, 594). Pembrolizumab plus chemotherapy is more effective than chemotherapy alone in treating patients with PD-L1–negative advanced or metastatic NSCLC, according to a pooled analysis of three randomized clinical trials (595). Patients with NSCLC who tested positive for PD-L1 showed similar outcomes. According to Zhang *et al.* (596), chemotherapy alone was not as effective as the combination regimen of toripalimab and pemetrexed/carboplatin. Furthermore, a combination of atezolizumab, bevacizumab, carboplatin, and paclitaxel significantly improved the median overall survival of chemotherapy-naïve NSCLC patients, according to the results of the IMpower150 trial (597). In addition, anticytotoxic T lymphocyte–associated protein 4 ipilimumab was tested as a first-line treatment for NSCLC in conjunction with nivolumab and platinum doublet chemotherapy (598).

## Conclusions and perspectives

There is always a need to develop novel therapeutic approaches. TCP enables therapeutic reprogramming and may even be able to reverse tissue attrition, despite its association with therapy escape. Here, we discussed related combination therapy plans and summarized the current therapeutic strategies that aim to block or reverse TCP (438).

It has been observed that directly imposing EMT-modulating agents or controlling plasticity-related TME with ICB does not significantly advance cancer treatment (438). However, every little bit helps in the fight against cancer, and TCP is a useful target for developing new treatment approaches. It is theoretically possible to inhibit unexpected TCP by maintaining tumor cell integrity (438, 599).

Efficient TCP manipulation shows promise. One treatment option might be to combine various tumor cells into a single, vulnerable form. It is important to keep using the basket trial design to investigate novel therapeutic approaches for various cancer types, with a focus on finding new targets to improve TCP manipulation (600). Modern technology might prove to be helpful in future TCP research. For example, the omics approach and capacity to manage “big data” have significantly increased the understanding of the molecular composition of tumor tissues. Multiple omics data can now be simultaneously captured at the single-cell level, and several analytical models have been developed to make sense of high-dimensional multimodal data (601).

In incredibly varied malignancies, single-cell multiomics may be able to map each individual cell’s response to treatment from transcriptomics, proteomics, and epigenomics perspectives, regardless of geographic distribution. New analytical methods provide comprehensive and in-depth data at the single-cell resolution and might aid in identifying new intracellular and intercellular cellular activity (506, 507). Additionally, the identification of TCP biomarkers, tumor responses to targeted therapy, and prognosis could be facilitated by single-cell techniques (602, 603).

Using artificial intelligence to identify underlying patterns in disjointed datasets is an additional option (604). It is anticipated that 1 day, whole cures might be obtained through individualized treatment plans determined by molecules at the single-cell level due to big data and its interpretive power. Cancer is an erratic, unpredictable, and dangerous illness (604). Therefore, scientific research will always attempt to reduce the complexity of the disease to a more manageable level (605). Research to better understand the role of TCP in targeted treatments may pave the way for future prevention of therapy evasion, which might result in long-lasting therapeutic benefits or a complete cure for cancer (605).

## Conflict of interest

The authors declare that have no conflicts of interest with the contents of this article.
